# Supplementary material for: Phylogenetic relationships in the southern African genus Drosanthemum (Ruschioideae, Aizoaceae)
Source: PeerJ. 2020 May 8;8:e8999. doi: 10.7717/peerj.8999 (PMC7213013; doi:10.7717/peerj.8999)
Supplement: Supplemental Information 1 [file peerj-08-8999-s001.pdf]

Supplementary information S1

to: Phylogenetic relationships in the southern African genus *Drosanthemum* (Ruschioideae, Aizoaceae)

by Liede-Schumann, Grimm, Nürk, Potts, Meve & Hartmann

Table S1: **Voucher**. Sequences newly produced for the present study are indicated in **bold**.

Species	Section	Voucher	Origin	ITS	rps16-trnK	trnQ-rps16	rpl16	trnS-G
Drosanthemum acuminatum L.Bolus	*Necopina*	Hartmann & Bayer 34608 (HBG)	South Africa: Western Cape-Montagu	HG324008	HG323956	HG323982	**LR030593**	**LR030877**
Drosanthemum acutifolium (L.Bolus) L.Bolus	*Drosanthemum*	Bruckmann & Hansen 32406 (HBG)	South Africa: Western Cape-Riversdale	**LR030506**	**LR030685**	**LR030783**	**LR030594**	**LR030878**
Drosanthemum albiflorum (L.Bolus) Schwantes	*Vespertina*	Bruckmann & Hansen 32383 (HBG)	South Africa: Western Cape-Oudtshoorn	---	**LR030686**	**LR030784**	**LR030595**	**LR030879**
Drosanthemum ambiguum L.Bolus	*Drosanthemum*	Hartmann 30388 (HBG)	South Africa: Western Cape-Bredasdorp	---	**LR030687**	**LR030785**	**LR030596**	**LR030880**
Drosanthemum anemophilum Van Jaarsveld & S.A.Hammer	*Decidua*	VanJaarsveld sn	South Africa: Western Cape-Laingsburg	LR030981	LR031140	LR031092	LR031041	LR031198
Drosanthemum archeri L.Bolus	*Drosanthemum*	Hartmann et al. 31774 (HBG)	South Africa: Northern Cape-Fraserburg	**LR030507**	**LR030688**	**LR030786**	**LR030597**	**LR030881**
Drosanthemum archeri L.Bolus	*Drosanthemum*	Hartmann et al. 31788 (HBG)	South Africa: Western Cape-Prince Albert	**LR030508**	**LR030689**	**LR030787**	**LR030598**	**LR030882**
Drosanthemum asperulum (Salm-Dyck) Schwantes	*Quadrata*	Bruyns 9005 (BOL)	South Africa: Western Cape-Montagu	---	---	KF131854	KF132147	KF133114

Species	Section	Voucher	Origin	ITS	rps16-trnK	trnQ-rps16	rpl16	trnS-G
Drosanthemum asperulum (Salm-Dyck) Schwantes	*Quadrata*	Hartmann & Bayer 34502 (HBG)	South Africa: Western Cape-Swellendam	LR030982	LR031141	LR031093	LR031042	LR031199
Drosanthemum attenuatum (Haw.) Schwantes	*Ossicula*	Hartmann & Liede 11826 (HBG)	South Africa: Western Cape-Simonstown	LR030983	LR031142	LR031094	LR031043	LR031200
Drosanthemum attenuatum (Haw.) Schwantes *aff.*	*Ossicula*	Hartmann & Court 34650 (HBG)	South Africa: Western Cape-Mosselbay	**LR030509**	**LR030690**	**LR030788**	**LR030599**	**LR030883**
Drosanthemum austricola L.Bolus	*Ossicula*	Hartmann & Bayer 34695 (HBG)	South Africa: Western Cape-Bredasdorp	**LR030510**	**LR030691**	**LR030789**	**LR030600**	**LR030884**
Drosanthemum bicolor L.Bolus	*Necopina*	Bruckmann & Hansen 32459 (HBG)	South Africa Western Cape-Worcester	**LR030511**	**LR030692**	**LR030790**	**LR030601**	**LR030885**
Drosanthemum boerhavii (Ecklon) H.E.K.Hartmann	*Speciosa*	Hartmann & Bayer 34697 (HBG)	South Africa: Western Cape-Swellendam	**LR030512**	**LR030693**	**LR030791**	**LR030602**	**LR030886**
Drosanthemum boerhavii (Ecklon) H.E.K.Hartmann (*D. aureopurpureum*)	*Speciosa*	Hartmann & Bayer 34478 (HBG)	South Africa: Western Cape-Swellendam	**LR030513**	**LR030694**	**LR030792**	---	---
Drosanthemum boerhavii (Ecklon) H.E.K.Hartmann (*D. insolitum*)	*Speciosa*	Hartmann & Bayer 34596 (HBG)	South Africa: Western Cape-Worcester	LR030984	LR031143	LR031095	LR031044	LR031201
Drosanthemum boerhavii (*D. strictifolium* L.Bolus)	*Speciosa*	Hartmann & Bayer 34480 (HBG)	South Africa: Western Cape-Heidelberg	**LR030514**	**LR030695**	**LR030793**	**LR030603**	**LR030887**
Drosanthemum brakfonteinense Liede, A.Schweiger & H.E.K.Hartmann	*Speciosa*	Hartmann 34688 (HBG)	South Africa: Western Cape-Swellendam	**LR030515**	**LR030696**	**LR030794**	**LR030604**	**LR030888**
Drosanthemum brevifolium (Aiton) Schwantes	*Drosanthemum*	Bruckmann & Hansen 32242 (HBG)	South Africa: Western Cape-Vanrhynsdorp	**LR030516**	**LR030697**	**LR030795**	**LR030605**	**LR030889**

Species	Section	Voucher	Origin	ITS	rps16-trnK	trnQ-rps16	rpl16	trnS-G
Drosanthemum calycinum (Haw.) Schwantes	*Quastea*	Bruckmann & Hansen 32211 (HBG)	South Africa: Western Cape-Malmesbury	HG324009	HG323957	HG323983	---	**LR030890**
Drosanthemum candens (Haw.) Schwantes	*Drosanthemum*	Hartmann & Dehn 25014 (HBG)	South Africa: Eastern Cape-Albany	LR030985	LR031144	LR031096	LR031045	LR031202
Drosanthemum candens (Haw.) Schwantes	*Drosanthemum*	Hartmann 30541 (HBG)	South Africa: Western Cape-Murraysburg	**LR030517**	**LR030698**	**LR030796**	**LR030606**	**LR030891**
Drosanthemum candens (Haw.) Schwantes	*Drosanthemum*	Hartmann et al. 31897 (HBG)	South Africa: Eastern Cape-Albany	**LR030518**	**LR030699**	**LR030797**	**LR030607**	**LR030892**
Drosanthemum cereale L.Bolus	*Speciosa*	Hartmann & Bayer 34490 (HBG)	South Africa: Western Cape-Caledon	---	**LR030700**	**LR030798**	---	---
Drosanthemum cereale L.Bolus	*Speciosa*	Hartmann & Bayer 34491 (HBG)	South Africa: Western Cape-Caledon	**LR030519**	**LR030701**	---	---	**LR030893**
Drosanthemum cereale L.Bolus	*Speciosa*	Hartmann & Bayer 34492 (HBG)	South Africa: Western Cape-Caledon	**LR030520**	**LR030702**	**LR030799**	**LR030608**	**LR030894**
Drosanthemum chrysum L.Bolus	*Speciosa*	Hartmann & Bayer 34631 (HBG)	South Africa: Western Cape-Caledon	HG324010	HG323958	HG323984	**LR030609**	**LR030895**
Drosanthemum crassum L.Bolus	*Vespertina*	Hartmann et al. 25896 (HBG)	South Africa: Western Cape-Swellendam	HG324011	HG323959	HG323985	**LR030610**	**LR030896**
Drosanthemum curtophyllum L.Bolus	*Xamera*	Hartmann & Potgieter 32673 (HBG)	South Africa: Northern Cape-Namaqualand	HG324012	HG323960	HG323986	**LR030611**	**LR030897**
Drosanthemum cymiferum L.Bolus	*Quastea*	Hartmann et al. 25811 (HBG)	South Africa: Northern Cape-Calvinia	**LR030521**	**LR030703**	**LR030800**	---	**LR030898**

**Species**	**Section**	**Voucher**	**Origin**	**ITS**	**rps16-trnK**	**trnQ-rps16**	**rpl16**	**trnS-G**
Drosanthemum cymiferum L.Bolus	*Quastea*	Hartmann et al. 31686 (HBG)	South Africa: Northern Cape-Calvinia	**LR030522**	**LR030704**	**LR030801**	**LR030612**	**LR030899**
Drosanthemum cymiferum L.Bolus	*Quastea*	Bruckmann & Hansen 32250 (HBG)	South Africa: Northern Cape-Calvinia	**LR030523**	**LR030705**	**LR030802**	---	**LR030900**
Drosanthemum deciduum H.E.K.Hartmann & Bruckmann	*Decidua*	Bruckmann & Hansen 32241 (HBG)	South Africa: Western Cape-Vanrhynsdorp	---	**LR030706**	**LR030803**	**LR030613**	**LR030901**
Drosanthemum deciduum H.E.K.Hartmann & Bruckmann	*Decidua*	Klak 1638 (BOL)	South Africa: Western Cape-Vanrhynsdorp	---	---	KF131855	KF132148	KF133115
Drosanthemum dejagerae L.Bolus	*Xamera*	Hartmann et al. 31812 (HBG)	South Africa: Western Cape-Prince Albert	**LR030524**	**LR030707**	**LR030804**	**LR030614**	**LR030902**
Drosanthemum delicatulum (L.Bolus) Schwantes	*Drosanthemum*	Hartmann 30782 (HBG)	South Africa: Western Cape-Swellendam	---	**LR030708**	**LR030805**	**LR030615**	**LR030903**
Drosanthemum delicatulum (L.Bolus) Schwantes	*Drosanthemum*	Bruckmann & Hansen 32462 (HBG)	South Africa: Western Cape-Worcester	LR030986	LR031145	LR031097	LR031046	LR031203
Drosanthemum dipageae H.E.K.Hartmann	*Xamera*	Hartmann 34409 (HBG)	South Africa: Eastern Cape-Uitenhage	**LR030525**	**LR030709**	**LR030806**	**LR030616**	**LR030904**
Drosanthemum ecclesianum Liede & H.E.K.Hartmann	*Necopina*	Hartmann 34814 (HBG)	South Africa: Western Cape-Uniondale	**LR030526**	**LR030710**	**LR030807**	**LR030617**	**LR030905**
Drosanthemum sp. ecclesianum Liede & H.E.K.Hartmann	*Necopina*	Hartmann 34815 (HBG)	South Africa: Western Cape-Uniondale	**LR030527**	**LR030711**	**LR030808**	**LR030618**	---
Drosanthemum edwardsiae L.Bolus	*Speciosa*	Hartmann & Court 34648 (HBG)	South Africa: Western Cape-Mosselbay	**LR030528**	**LR030712**	**LR030809**	**LR030619**	---

Species	Section	Voucher	Origin	ITS	rps16-trnK	trnQ-rps16	rpl16	trnS-G
Drosanthemum edwardsiae L.Bolus	*Speciosa*	Hartmann & Court 34651 (HBG)	South Africa: Western Cape-Mosselbay	HG324014	HG323962	HG323988	LR031047	---
Drosanthemum erigeriflorum (Jacq.) Stearn	*Drosanthemum*	Hartmann & Dehn 26196 (HBG)	South Africa: Western Cape-Heidelberg	**LR030529**	**LR030713**	**LR030810**	**LR030620**	**LR030906**
Drosanthemum expersum (N.E.Br.) Schwantes	*Quastea*	Hartmann & Bayer 34590 (HBG)	South Africa: Western Cape-Ceres	**LR030530**	**LR030714**	**LR030811**	**LR030621**	**LR030907**
Drosanthemum expersum (N.E.Br.) Schwantes	*Quastea*	Hartmann & Bayer 34598 (HBG)	South Africa: Western Cape-Montagu	**LR030579**	**LR030766**	**LR030861**	**LR030667**	**LR030961**
Drosanthemum flammeum L.Bolus	*Speciosa*	Hartmann & Bayer 34460 (HBG)	South Africa: Western Cape-Worcester	HG324015	HG323963	HG323989	---	---
Drosanthemum flavum (Haw.) Schwantes	*Speciosa*	Hartmann & Bayer 34706 (HBG)	South Africa: Western Cape-Caledon	**LR030531**	**LR030715**	**LR030812**	**LR030622**	**LR030908**
Drosanthemum floribundum (Haw.) Schwantes cf.	*Drosanthemum*	Hartmann 34403 (HBG)	South Africa: Eastern Cape-Port Elizabeth	**LR030532**	**LR030716**	**LR030813**	**LR030623**	**LR030909**
Drosanthemum fourcadei Schwantes	*Xamera*	Hartmann 33900 (HBG)	South Africa: Eastern Cape-Uitenhage	**LR030533**	**LR030717**	**LR030814**	**LR030624**	**LR030910**
Drosanthemum fourcadei Schwantes	*Xamera*	Hartmann 34404 (HBG)	South Africa: Eastern Cape-Port Elizabeth	**LR030552**	**LR030736**	**LR030832**	**LR030638**	**LR030929**
Drosanthemum framesii L.Bolus	*Drosanthemum*	Bruckmann & Hansen 32339 (UBT)	South Africa: Western Cape-Ceres	**LR030534**	**LR030718**	**LR030815**	**LR030625**	**LR030911**
Drosanthemum glabrescens L. Bolus	*Drosanthemum*	Bruckmann & Hansen 32256 (UBT)	South Africa: Northern Cape-Calvinia	**LR030535**	**LR030719**	**LR030816**	**LR030626**	**LR030912**
Drosanthemum gracillimum L.Bolus	*Vespertina*	Bruyns 7170 (BOL)	South Africa: Western Cape-Robertson	---	---	KF131857	---	KF133117

Species	Section	Voucher	Origin	ITS	rps16-trnK	trnQ-rps16	rpl16	trnS-G
Drosanthemum hallii L.Bolus	*Speciosa*	Hartmann & Bayer 34610 (HBG)	South Africa: Western Cape-Worcester	**LR030536**	**LR030720**	**LR030817**	---	**LR030913**
Drosanthemum hispidum (Haw.) Schwantes	*Drosanthemum*	Hartmann 33866 (HBG)	South Africa: Eastern Cape-Port Elizabeth	HG324016	HG323964	HG323990	LR031048	LR031204
Drosanthemum hispidum (Haw.) Schwantes	*Drosanthemum*	Hartmann 34262 (HBG)	South Africa: Eastern Cape-Port Elizabeth	**LR030537**	**LR030721**	**LR030818**	**LR030627**	**LR030914**
Drosanthemum hispifolium (Haw.) Schwantes	*Ossicula*	Bruckmann & Hansen 32206 (HBG)	South Africa: Western Cape-Malmesbury	HG324017	HG323965	HG323991	LR031049	LR031205
Drosanthemum hispifolium (Haw.) Schwantes	*Ossicula*	Hartmann & Bayer 34587 (HBG)	South Africa: Western Cape-Tulbagh	**LR030538**	**LR030722**	**LR030819**	**LR030628**	**LR030915**
Drosanthemum inornatum (L.Bolus) L.Bolus	*Decidua*	Bruyns 10066 (BOL)	Namibia: Rosh Pinah	---	---	KF131858	KF132150	KF133118
Drosanthemum inornatum (L.Bolus) L.Bolus	*Decidua*	Hartmann & Potgieter 32654 (HBG)	South Africa: Northern Cape-Namaqualand-Numees	**LR030539**	**LR030723**	**LR030820**	---	**LR030916**
Drosanthemum intermedium L.Bolus		Bruckmann & Hansen 32200 (HBG)	South Africa: Western Cape-Simonstown	LR030987	LR031146	LR031098	LR031050	LR031206
Drosanthemum intermedium L.Bolus		Bruckmann & Hansen 32439 (HBG)	South Africa: Western Cape-Bredasdorp	**LR030540**	**LR030724**	**LR030821**	**LR030629**	**LR030917**
Drosanthemum latipetalum L.Bolus	*Drosanthemum*	Hartmann et al. 31568 (HBG)	South Africa: Northern Cape: Namaqualand	**LR030541**	**LR030725**	**LR030822**	**LR030630**	**LR030918**
Drosanthemum latipetalum L.Bolus	*Drosanthemum*	Hartmann et al. 31605 (HBG)	South Africa: Northern Cape: Namaqualand	**LR030542**	**LR030726**	**LR030823**	**LR030631**	**LR030919**

Species	Section	Voucher	Origin	ITS	rps16-trnK	trnQ-rps16	rpl16	trnS-G
Drosanthemum latipetalum L.Bolus	*Drosanthemum*	Bruckmann & Hansen 32217 (HBG)	South Africa: Western Cape-Clanwilliam	**LR030543**	**LR030727**	**LR030824**	**LR030632**	**LR030920**
Drosanthemum lavisii L.Bolus	*Speciosa*	Hartmann & Bayer 34693 (HBG)	South Africa: Western Cape-Napier	**LR030544**	**LR030728**	**LR030825**	**LR030633**	**LR030921**
Drosanthemum lique (N.E.Br.) Schwantes	*Vespertina*	Hartmann 34447 (HBG)	South Africa: Northern Cape-Williston	HG324018	HG323966	HG323992	---	**LR030922**
Drosanthemum longipes (L.Bolus) H.E.K.Hartmann	*Decidua*	Bruyns 6037 (BOL)	South Africa: Northern Cape-Calvinia	---	---	KF131859	KF132151	KF133119
Drosanthemum longipes (L.Bolus) H.E.K.Hartmann	*Decidua*	Van Jaarsveld s.n. (HBG)	South Africa: Western Cape-Riversdale	LR030988	LR031147	LR031099	LR031051	LR031207
Drosanthemum luederitzii (Engler) Schwantes	*Drosanthemum*	Hartmann et al. 26095 (HBG)	Namibia: Lüderitz-Süd	**LR030546**	**LR030730**	**LR030827**	**LR030635**	**LR030924**
Drosanthemum marinum L. Bolus	*Drosanthemum*	Bruckmann & Hansen 32205 (HBG)	South Africa: Western Cape-Malmesbury	**LR030547**	**LR030731**	**LR030828**	**LR030636**	**LR030925**
Drosanthemum micans (L.) Schwantes	*Speciosa*	Hartmann & Bayer 34597 (HBG)	South Africa: Western Cape-Montagu	**LR030548**	**LR030732**	**LR030829**	---	**LR030926**
Drosanthemum micans (L.) Schwantes	*Speciosa*	LeRoux 83/2 (HBG)	South Africa: Western Cape-Worcester	**LR030549**	**LR030733**	**LR030830**	---	**LR030927**
Drosanthemum muirii L.Bolus	*Drosanthemum*	Hartmann 30321 (HBG)	South Africa: Western Cape-Laingsburg	**LR030551**	**LR030735**	**LR030831**	**LR030637**	**LR030928**
Drosanthemum muirii L.Bolus	*Drosanthemum*	Bruckmann & Hansen 32209 (HBG)	South Africa: Northern Cape-Calvinia	**LR030550**	**LR030734**	---	---	---
Drosanthemum nollothense Liede & H.E.K.Hartmann	*Drosanthemum*	Hartmann 31547 (HBG)	South Africa: Northern Cape-Namaqualand	**LR030553**	**LR030737**	**LR030833**	**LR030639**	**LR030930**

Species	Section	Voucher	Origin	ITS	rps16-trnK	trnQ-rps16	rpl16	trnS-G
Drosanthemum nollothense Liede & H.E.K.Hartmann	*Drosanthemum*	Hartmann et al. 31569 (HBG)	South Africa: Northern Cape-Namaqualand	**LR030554**	**LR030738**	**LR030834**	**LR030640**	**LR030931**
Drosanthemum nordenstamii L.Bolus	*Drosanthemum*	Hartmann et al. 31533 (HBG)	South Africa: Northern Cape-Namaqualand	**LR030555**	**LR030739**	**LR030835**	**LR030641**	**LR030932**
Drosanthemum obibense Liede & H.E.K.Hartmann	*Drosanthemum*	Hartmann et al. 25972 (HBG)	Namibia: Lüderitz-Süd-Diamanten-Sperrgebiet	**LR030545**	**LR030729**	**LR030826**	**LR030634**	**LR030923**
Drosanthemum obibense Liede & H.E.K.Hartmann	*Drosanthemum*	Hartmann et al. 25979 (HBG)	Namibia: Lüderitz-Süd	**LR030556**	**LR030740**	**LR030836**	**LR030642**	**LR030933**
Drosanthemum oculatum L.Bolus	*Drosanthemum*	Hartmann et al. 31680 (HBG)	South Africa: Northern Cape-Calvinia	**LR030557**	**LR030741**	**LR030837**	**LR030643**	**LR030934**
Drosanthemum oculatum L.Bolus	*Drosanthemum*	Hartmann et al. 31710 (HBG)	South Africa: Northern Cape-Calvinia	**LR030558**	**LR030742**	**LR030838**	---	**LR030935**
Drosanthemum opacum L.Bolus	*Drosanthemum*	Bruckmann & Hansen 32212 (HBG)	South Africa: Western Cape-Morreesburg	**LR030559**	**LR030743**	**LR030839**	**LR030644**	**LR030936**
Drosanthemum pallens (Haw.) Schwantes (*D. stokoei*)	*Ossicula*	Bayer 7454 (HBG)	South Africa: Northern Cape: Namaqualand	HG324021	HG323970	HG323996	**LR030645**	**LR030937**
Drosanthemum papillatum L.Bolus	*Quastea*	Hartmann 34456 (HBG) - Worcester	South Africa: Western Cape-Worcester	**LR030560**	**LR030744**	**LR030840**	**LR030646**	**LR030938**
Drosanthemum papillatum L.Bolus	*Quastea*	Hartmann & Bayer 34607 (HBG)	South Africa: Western Cape-Swellendam	HG324020	HG323968	HG323994	LR031052	LR031208
Drosanthemum papillatum L.Bolus	*Quastea*	Hartmann & Bayer 34614 (HBG)	South Africa: Western Cape-Worcester	**LR030561**	**LR030745**	**LR030841**	**LR030647**	**LR030939**

Species	Section	Voucher	Origin	ITS	rps16-trnK	trnQ-rps16	rpl16	trnS-G
Drosanthemum papillatum L.Bolus	*Quastea*	Hartmann & Bayer 34624 (HBG)	South Africa: Western Cape-Montagu	**LR030562**	**LR030746**	**LR030842**	**LR030648**	**LR030940**
Drosanthemum papillatum L.Bolus	*Quastea*	Hartmann & Bayer 34629 (HBG)	South Africa: Western Cape-Caledon	---	**LR030747**	**LR030843**	---	**LR030941**
Drosanthemum parvifolium (Haw.) Schwantes	*Drosanthemum*	Hartmann 30399 (HBG)	South Africa: Western Cape-Bredasdorp	**LR030563**	**LR030748**	**LR030844**	**LR030649**	**LR030942**
Drosanthemum praecultum (N.E.Br.) Schwantes (*D. montaguense*)	*Xamera*	Hartmann et al. 31824 (HBG)	South Africa: Western Cape-Uniondale	HG324019	HG323967	HG323993	**LR030650**	**LR030943**
Drosanthemum prostratum L.Bolus *aff.*	*Drosanthemum*	Hartmann 34316 (HBG)	South Africa: Northern Cape-Calvinia	**LR030564**	**LR030749**	**LR030845**	**LR030651**	**LR030944**
Drosanthemum prostratum L.Bolus	*Drosanthemum*	Hartmann & Bayer 34592 (HBG)	South Africa: Western Cape-Ceres	**LR030565**	**LR030750**	**LR030846**	**LR030652**	**LR030945**
Drosanthemum pulchrum L.Bolus	*Speciosa*	Hartmann & Bayer 34712 (HBG)	South Africa: Western Cape-Worcester	**LR030566**	**LR030751**	**LR030847**	---	**LR030946**
Drosanthemum quadratum Klak	*Quadrata*	Bruyns 7812 (BOL)	South Africa: Western Cape-Swellendam	---	---	KF131861	KF132153	KF133121
Drosanthemum quadratum Klak	*Quadrata*	Hartmann & Bayer 34503 (HBG)	South Africa: Western Cape-Swellendam	**LR030568**	**LR030753**	**LR030849**	**LR030654**	**LR030948**
Drosanthemum ramosissimum L.Bolus	*Drosanthemum*	Schmiedel 110492 (HBG)	South Africa: Western Cape-Moedverloren	**LR030569**	**LR030754**	**LR030850**	**LR030655**	**LR030949**
Drosanthemum schoenlandianum L.Bolus	*Drosanthemum*	Bruyns 7172 (BOL)		AJ438214	---	JN896435	KF132154	JN896382
Drosanthemum schoenlandianum L.Bolus	*Drosanthemum*	Hartmann et al. 25751 (HBG)	South Africa: Northern Cape-Calvinia	LR030989	LR031148	LR031100	LR031053	---

Species	Section	Voucher	Origin	ITS	rps16-trnK	trnQ-rps16	rpl16	trnS-G
Drosanthemum semiglobosum L.Bolus	*Necopina*	Hartmann & Bayer 34593 (HBG)	South Africa: Western Cape-Worcester	**LR030570**	**LR030755**	**LR030851**	**LR030656**	**LR030950**
Drosanthemum sp.	*Drosanthemum*	Bruckmann & Hansen 32218 (HBG)	South Africa: Western Cape-Clanwilliam	**LR030571**	**LR030756**	**LR030852**	**LR030657**	**LR030951**
Drosanthemum sp.	*Drosanthemum*	Bruckmann & Hansen 32382 (HBG)	South Africa: Western Cape-Oudtshoorn	---	**LR030757**	**LR030853**	**LR030658**	**LR030952**
Drosanthemum sp.	*Drosanthemum*	Bruckmann & Hansen 32392 (HBG)	South Africa: Western Cape-Mosselbay	**LR030572**	**LR030758**	**LR030854**	**LR030659**	**LR030953**
Drosanthemum sp.	*Drosanthemum*	Hartmann & Bayer 34472 (HBG)	South Africa: Western Cape-Robertson	**LR030573**	**LR030759**	**LR030855**	**LR030660**	**LR030954**
Drosanthemum sp.	*Drosanthemum*	Hartmann & Bayer 34496 (HBG)	South Africa: Western Cape-Swellendam	**LR030574**	**LR030760**	**LR030856**	**LR030661**	**LR030955**
Drosanthemum sp.	*Drosanthemum*	Hartmann 30568 (HBG)	South Africa: Eastern Cape-Graaff-Reinet	**LR030575**	**LR030761**	**LR030857**	**LR030662**	**LR030956**
Drosanthemum sp.	*Drosanthemum*	Hartmann 33182 (HBG)	South Africa: Northern Cape-De Aar	**LR030576**	**LR030762**	**LR030858**	**LR030663**	**LR030957**
Drosanthemum sp.	*Drosanthemum*	Hartmann et al. 25365 (HBG)	South Africa: Western Cape-Prince Albert	**LR030577**	**LR030763**	**LR030859**	**LR030664**	**LR030958**
Drosanthemum sp.	*Drosanthemum*	Hartmann et al. 25376 (HBG)	South Africa: Western Cape-Ceres	---	**LR030764**	**LR030860**	**LR030665**	**LR030959**
Drosanthemum sp.	*Ossicula*	Hartmann & Bayer 34699 (HBG)	South Africa: Western Cape-Swellendam	**LR030578**	**LR030765**	**LR215984**	**LR030666**	**LR030960**

Species	Section	Voucher	Origin	ITS	rps16-trnK	trnQ-rps16	rpl16	trnS-G
Drosanthemum sp.	*Vespertina*	Bruckmann & Hansen 32378 (HBG)	South Africa: Western Cape-Prince Albert	**LR030580**	**LR030767**	**LR030862**	**LR030668**	**LR030962**
Drosanthemum sp.	*Vespertina*	Bruckmann & Hansen 32404 (HBG)	South Africa: Western Cape-Riversdale	**LR030581**	**LR030768**	**LR030863**	**LR030669**	**LR030963**
Drosanthemum sp.	*Vespertina*	Hartmann 31059 (HBG)	South Africa: Northern Cape-Philipstown	**LR030582**	**LR030769**	**LR030864**	**LR030670**	**LR030964**
Drosanthemum sp.	*Vespertina*	Hartmann 34804 (HBG)	South Africa: Eastern Cape-Port Elizabeth	**LR030583**	**LR030770**	**LR030865**	**LR030671**	**LR030965**
Drosanthemum sp.	*Vespertina*	Hartmann et al. 25424 (HBG)	South Africa: Northern Cape-Namaqualand	---	**LR030771**	**LR030866**	**LR030672**	**LR030966**
Drosanthemum sp.	*Vespertina*	Hartmann et al. 26170a (HBG)	South Africa: Western Cape-Vredendal	**LR030584**	**LR030772**	**LR030867**	**LR030673**	**LR030967**
Drosanthemum sp.	*Vespertina*	Hartmann et al. 31925 (HBG)	South Africa: Eastern Cape-Cradock	---	**LR030773**	**LR030868**	**LR030674**	**LR030968**
Drosanthemum sp.	*Vespertina*	Hartmann et al. 33490 (HBG)	South Africa: Eastern Cape-Hofmeyr	**LR030585**	**LR030774**	**LR030869**	**LR030675**	**LR030969**
Drosanthemum sp.	*Xamera*	Hartmann & Milton 34586 (HBG)	South Africa: Western Cape-Prince Albert	**LR030586**	**LR030775**	**LR030870**	**LR030676**	**LR030970**
Drosanthemum sp.	*Xamera*	Hartmann 34424 (HBG)	South Africa: Northern Cape-Victoria West	**LR030587**	**LR030776**	**LR030871**	**LR030677**	**LR030971**
Drosanthemum sp.	*Drosanthemum*	Mucina 161005-14 (NBG)	South Africa: Western Cape-Piketberg	**LR030588**	**LR030777**	**LR030872**	**LR030678**	**LR030972**

**Species**	**Section**	**Voucher**	**Origin**	**ITS**	**rps16-trnK**	**trnQ-rps16**	**rpl16**	**trnS-G**
Drosanthemum speciosum (Haw.) Schwantes	*Speciosa*	Bruyns 9006 (BOL)	South Africa: Western Cape-Worcester	---	---	KF131862	KF132155	KF133122
Drosanthemum speciosum (Haw.) Schwantes	*Speciosa*	Hartmann & Bayer 34619 (HBG)	South Africa: Western Cape-Worcester	HE585039	HG323969	HG323995	---	---
Drosanthemum striatum Schwantes	*Ossicula*	Hartmann 29013 (HBG)	South Africa: Western Cape-Worcester	**LR030589**	**LR030778**	---	**LR030679**	**LR030973**
Drosanthemum striatum Schwantes	*Ossicula*	Hartmann & Bayer 34698 (HBG)	South Africa: Western Cape-Swellendam	**LR030567**	**LR030752**	**LR030848**	**LR030653**	**LR030947**
Drosanthemum striatum Schwantes	*Ossicula*	Hartmann & Bayer 34720 (HBG)	South Africa: Western Cape-Worcester	**LR030590**	**LR030779**	**LR030873**	**LR030680**	**LR030974**
Drosanthemum subclausum L.Bolus	*Drosanthemum*	Hartmann et al. 25737 (HBG)	South Africa: Western Cape-Vanrhynsdorp	HG324022	HG323971	HG323997	**LR030681**	**LR030975**
Drosanthemum subplanum L.Bolus	*Drosanthemum*	Bruckmann & Hansen 32259 (HBG)	South Africa: Northern Cape-Calvinia	**LR030591**	**LR030780**	**LR030874**	**LR030682**	**LR030976**
Drosanthemum tetramerum H.E.K.Hartmann	*Quadrata*	Hartmann & Bayer 34488 (HBG)	South Africa: Western Cape-Caledon	LR030990	LR031149	LR031101	---	---
Drosanthemum thudichumii L.Bolus	*Necopina*	Hartmann & Bayer 34714 (HBG)	South Africa: Western Cape-Worcester	HG324023	HG323972	HG323998	LR031054	LR031209
Drosanthemum tuberculiferum L. Bolus	*Drosanthemum*	Bruckmann & Hansen 32398 (HBG)	South Africa: Western Cape-Riversdale	---	**LR030781**	**LR030875**	**LR030683**	**LR030977**
Drosanthemum uniondalense H.E.K.Hartmann	*Speciosa*	Hartmann 34813 (HBG)	South Africa: Western Cape-Uniondale	**LR030592**	**LR030782**	**LR030876**	**LR030684**	**LR030978**

Species	Section	Voucher	Origin	ITS	rps16-trnK	trnQ-rps16	rpl16	trnS-G
Drosanthemum zygophylloides (L.Bolus) L.Bolus		Klak 830 (BOL)	South Africa: Western Cape-Piketberg	---	---	KF131863	KF132156	KF133123
Drosanthemum zygophylloides (L.Bolus) L.Bolus		Mucina 130216-4	South Africa: Western Cape-Piketberg	LR030991	LR031150	LR031102	LR031055	LR031210
**Outgroup**	**Clade in Klak et al. (2013)**							
Antegibbaeum fissoides (Haw.) Schwantes ex H.Wulff	L3	Klak 308 (BOL)	South Africa: Western Cape	AJ438227	--	KF131867	KF132159	KF133127
Antimima ventricosa (L.Bolus) H.E.K.Hartmann	L2	Klak 475 (BOL)	South Africa: Northern Cape	AJ438225	--	JN896434	KF132163	JN896381
Braunsia geminata (Haw.) L.Bolus	L2	Klak 205 (BOL)	South Africa	AJ438228	--	KF131875	KF132168	KF133135
Carpobrotus muirii (L.Bolus) L.Bolus	L1	Klak 706 (BOL)	South Africa: Western Cape	AJ438230	--	KF131877	KF132170	KF133137
Cephalophyllum inaequale L.Bolus	L1	Hartmann 7883 (HBG)	South Africa: Western Cape	HG324002	HG323950	HG323976	LR031056	LR031211
Chasmatophyllum musculinum (Haw.) Dinter & Schwantes	L3	Bolduan s.n.	ex hort. ZSS (994202/0)	HG324003	HG324003	HG323977	LR031057	LR031212
Cheiridopsis pearsonii N.E.Br.	I	Bruyns 9504 (BOL)	South Africa	--	--	KF131885	KF132178	KF133145
Cheiridopsis rostrata (L.) N.E.Br.	I	Powell 88 (NBG)	South Africa: Western Cape	--	--	KY635278	KY635037	KY635358
Conophytum calculus (A.Berger) N.E.Br.	I	Klak 1641 (BOL)	South Africa: Western Cape	--	--	KF131887	KF132180	KF133147
		Ritz s.n.	ex hort.	FN386499	--	--	--	--
Corpuscularia lehmannii (Eckl. & Zeyh.) Schwantes	F	Klak 353 (BOL)	South Africa	--	--	KF131889	KF132182	KF133149
Corpuscularia lehmannii (Eckl. & Zeyh.) Schwantes		Hartmann & Kremling 33693 (HBG)	South Africa: Eastern Cape	AJ582942	--	--	--	--
Deilanthe peersii (L.Bolus) N.E.Br.	L4	Klak 1694 (BOL)	South Africa: Western Cape	--	--	KF131891	KF132184	KF133151
Deilanthe peersii (L.Bolus) N.E.Br.		Hartmann & Dehn 15486 (HBG)	South Africa: Eastern Cape	LR030992	LR031151	--	--	--

Species	Section	Voucher	Origin	ITS	rps16-trnK	trnQ-rps16	rpl16	trnS-G
Delosperma echinatum (Lam.) Schwantes	F	Hartmann & Kremling 33682 (HBG)	South Africa: Eastern Cape	HG324005	HG323953	HG323979	**LR031067**	**LR031225**
Delosperma esterhuyseniae L.Bolus	F	Bruyns 7141 (BOL)	South Africa	AJ438213	--	JN896438	KF132187	JN896385
Dicrocaulon brevifolium N.E.Br.	B	Hartmann 1480 (HBG)	South Africa: Western Cape	LR030993	LR031152	LR031103	LR031058	LR031213
Dicrocaulon microstigma (L.Bolus) Ihlenf.	B	Ihlenfeldt & Hartmann 4529 (HBG)	South Africa: Western Cape	HG324007	HG323955	HG323981	LR031059	LR031214
Disphyma dunsdonii L.Bolus	C	Klak 808 (BOL)	South Africa	--	--	KF131902	KF132194	KF133162
Disphyma dunsdonii L.Bolus		Hartmann & Bayer 34630 (HBG)	South Africa: Western Cape	LR030994	LR031153	--	--	--
Drosanthemopsis diversifolia (L.Bolus) Klak	H	Hartmann et al. 26169 (HBG)	South Africa: Western Cape	HG324013	HG323961	HG323987	LR031060	LR031215
Drosanthemopsis diversifolia (L.Bolus) Klak	H	Klak 1743 (BOL)	South Africa	--	--	KF131856	KF132149	KF133116
Drosanthemopsis vaginata (L.Bolus) Rauschert	H	Wisura 882 (BOL)	South Africa	--	--	KF131923	KF132215	KF133184
"Drosanthemum" pulverulentum (Haw.) Schwantes	L1	Klak 1847 (BOL)	South Africa: Western Cape	--	--	KF131860	KF132152	KF133120
Erepsia inclaudens (Haw.) Schwantes	L1	Bruyns 6847 (BOL)	South Africa: Western Cape	AJ438234	--	KF131907	KF132200	KF133168
Enarganthe octonaria (L.Bolus) N.E.Br.	I	Klak 491 (BOL)	South Africa: Northern Cape	--	--	JN896433	KF132198	JN896380
Enarganthe octonaria (L.Bolus) N.E.Br.		Hartmann 7505 (HBG)	South Africa: Northern Cape	LR030995	LR031154	--	--	--
Esterhuysenia mucronata (L.Bolus) Klak	L2	Klak 709 (BOL)	South Africa: Western Cape	AJ438255		KF131908	KF132201	KF133169
Gibbaeum hortenseae (N.E.Br.) Thiede & Klak	E	Klak 1979 (BOL)	South Africa	--	--	KF131913	KF132205	KF133174
Gibbaeum pachypodium (Kensit) L.Bolus	E	Klak 380 (BOL)	South Africa	--	--	KF131914	KF132206	KF133175
Glottiphyllum cruciatum (Haw.) N.E.Br.	D	Bruyns 8207	South Africa	--	--	KF131915	KF132207	KF133176

Species	Section	Voucher	Origin	ITS	rps16-trnK	trnQ-rps16	rpl16	trnS-G
Glottiphyllum cruciatum (Haw.) N.E.Br.		Hartmann 8809 (HBG)	South Africa: Western Cape	LR030996	LR031155	--	--	--
Hartmanthus pergamentaceus (L.Bolus) S.A.Hammer	K	Klak 243 (BOL)	Namibia	--	--	KF131919	KF132211	KF133180
Hartmanthus pergamentaceus (L.Bolus) S.A.Hammer	K	Klak 1992 (BOL)		--	--	KF131920	KF132212	KF133181
Ihlenfeldtia excavata (L.Bolus) H.E.K.Hartmann	I	Wisura 1926 (BOL)	South Africa	--	--	KF131921	KF132214	KF133182
Ihlenfeldtia excavata (L.Bolus) H.E.K.Hartmann		Hartmann et al. 20587 (HBG)	South Africa: Northern Cape	LR030997	LR031156	--	--	--
Jacobsenia hallii L.Bolus	J	Ihlenfeldt & Hartmann 4463 (HBG)	South Africa: Northern Cape	HG324024	HG323973	HG323999	LR031061	LR031216
Jacobsenia kolbei (L.Bolus) L.Bolus & Schwantes	J	Klak 1819 (BOL)	South Africa	--	--	KF131922	--	KF133183
Jacobsenia kolbei (L.Bolus) L.Bolus & Schwantes		Ihlenfeldt & Hartmann 5060 (HBG)	South Africa: Western Cape	AJ582951	--	--	--	--
Lampranthus bicolor (L.) N.E.Br.	L1	Klak 543 (BOL)	South Africa: Western Cape	AJ438250	--	JN896437	KF132221	JN896384
Lithops julii (Dinter & Schwantes) N.E.Br. ssp. fulleri (N.E.Br.) Fearn	L1	Klak 701 (BOL)	South Africa: Northern Cape	AJ438218	--	KF131934	KF132227	KF133195
Malephora lutea (Haw) Schwantes	D	Klak 664 (BOL)	South Africa: Western Cape	--	--	KF131936	KF132229	KF133197
Meyerophytum globosum (L.Bolus) Ihlenf.	G	Hammer sub HBG 6314 (BOL)	South Africa	--	--	KF131939	KF132087	KF133200
Meyerophytum meyeri (Schwantes) Schwantes	G	Rawe s.n. (BOL)	South Africa	--	--	KF131940	KF132233	KF133201
Meyerophytum meyeri (Schwantes) Schwantes		Ihlenfeldt & Hartmann 5046 (HBG)	South Africa: Northern Cape	LR030998	LR031157	--	--	--
Mitrophyllum clivorum (N.E.Br.) Schwantes	G	Klak 1987 (BOL)	South Africa	--	--	KF131941	KF132234	KF133202

Species	Section	Voucher	Origin	ITS	rps16-trnK	trnQ-rps16	rpl16	trnS-G
Mitrophyllum clivorum (N.E.Br.) Schwantes		Ihlenfeldt & Hartmann 4681 (HBG)	South Africa: Northern Cape	LR030999	LR031158	--	--	--
Monilaria moniliformis (Thunb.) Ihlenf. & Jürgens	B	Klak 787 (BOL)	South Africa: Western Cape	--	--	KF131942	KF132235	KF133203
Monilaria moniliformis (Thunb.) Ihlenf. & Jürgens		Ihlenfeldt & Hartmann 4213 (HBG)	South Africa: Western Cape	LR031000	LR031159	--	--	--
Nananthus aloides (Haw.) Schwantes	L4	Klak 1797 (BOL)	South Africa: North West Province	--	--	KF131946	KF132238	KF133207
Nananthus aloides (Haw.) Schwantes		Hartmann 32029 (HBG)	South Africa: Northern Cape	LR031001	LR031160	--	--	--
Odontophorus marlothii N.E.Br.	I	Hartmann 7582 (HBG)	South Africa: Northern Cape	HG324025	HG323974	HG324000	LR031062	LR031217
Odontophorus marlothii N.E.Br.	I	Klak 862 (BOL)	South Africa: Northern Cape	--	--	KF131951	KF132243	KF133212
Oscularia deltoides (L.) Schwantes	L1	Klak 215 (BOL)	South Africa	AJ438215	--	KF131955	KF132246	KF133216
Pleiospilos simulans (Marloth) N.E.Br.	L4	Bruyns 4988 (BOL)	South Africa: Eastern Cape	--	--	KF131959	KF132250	KF133220
Polymita steenbokensis H.E.K.Hartmann	I	Bruyns 8267 (BOL)	South Africa: Northern Cape	--	--	KF131960	KF132251	KF133221
Polymita steenbokensis H.E.K.Hartmann		Hartmann & Bayer 78032 (HBG)	South Africa: Northern Cape	LR031002	LR031161	--	--	--
Prepodesma orpenii (N.E.Br.) N.E.Br.	L4	Klak 1800 (BOL)	South Africa: North West Province	--	--	KF131961	--	KF133222
Prepodesma orpenii (N.E.Br.) N.E.Br.		Hartmann & Dehn 19666 (HBG)	South Africa: Northern Cape	LR031003	LR031162	--	--	--
Roosia grahambeckii (Van Jaarsv.) Van Jaarsv.	--	VanJaarsveld s.n. (NBG)	South Africa: Western Cape	LR031004	LR031163	LR031104	LR031063	LR031218
Ruschia maxima (Haw.) L.Bolus	L1	Klak 704 (BOL)	South Africa	AJ438222	--	KF131969	KF132258	KF133231
Schlechteranthus hallii L.Bolus	I	Klak 259 (BOL)	South Africa: Northern Cape	--	--	KF131980	KF132268	KF133242

Species	Section	Voucher	Origin	ITS	rps16-trnK	trnQ-rps16	rpl16	trnS-G
Schlechteranthus hallii L.Bolus		Hartmann 8276 (HBG)	South Africa: Northern Cape	LR031005	--	--	--	--
Scopelogena bruynsii Klak	L1	Klak 462 (BOL)	South Africa: Western Cape	AJ438258	--	KF131982	KF132269	KF133244
Smicrostigma viride (Haw.) N.E.Br.	L2	Klak 180 (BOL)	South Africa: Western Cape	AJ438259	--	JN896432	KF132270	JN896379
Vlokia ater S.A.Hammer	L3	Hammer & Vlok 1181 (BOL)	South Africa: Western Cape	AJ438260	--	KF131992	KF132279	KF133254
Wooleya farinosa (L.Bolus) L.Bolus	L1	Hartmann 7213 (HBG)	South Africa: Northern Cape	HG324026	HG323975	HG324001	LR031064	LR031219
Zeuktophyllum suppositum (L.Bolus) N.E.Br.	L2	Klak 375 (BOL)	South Africa: Western Cape	AJ438262	--	KF131994	KF132281	KF133256
**Dorotheantheae**								
Cleretum bellidiforme (Burm.f.) G.D.Rowley		Klak 627 (BOL)/ Klak 1534 (BOL)	South Africa: South Western Cape	KF132145	JN896383	JN896436	--	JN896503
Cleretum papulosum (L.f.) L.Bolus		Klak 1487 (BOL)	South Africa: Northern Cape	KF132146	JN896408	JN896461	--	JN896507
Cleretum rourkei L. Bolus		Klak 1524 (BOL)	South Africa: Northern Cape	--	JN896500	JN896454	--	JN896500
**Apatesieae**								
Conicosia pugioniformis (L.) N.E.Br. ssp. *muirii* (N.E.Br.) Ihlenf. & Gerbaulet		Klak 1570	South Africa: South Western Cape	KF132144	JN896409	JN896462	--	JN896508
Conicosia pugioniformis (L.) N.E.Br.		Juergens sn.	South Africa	HG324004	HG323952	HG323978	--	--
