# Supplementary material for: Phylogenetic relationships in the southern African genus Drosanthemum (Ruschioideae, Aizoaceae)
Source: PeerJ. 2020 May 8;8:e8999. doi: 10.7717/peerj.8999 (PMC7213013; doi:10.7717/peerj.8999)
Supplement: Supplemental Information 2 [file peerj-08-8999-s002.pdf]

Supplementary information S2

to: Phylogenetic relationships in the southern African genus *Drosanthemum* (Ruschioideae, Aizoaceae)

by Liede-Schumann, Grimm, Nürk, Potts, Meve & Hartmann

Examples of character re-coding used for intra-clade haplotype analyses:

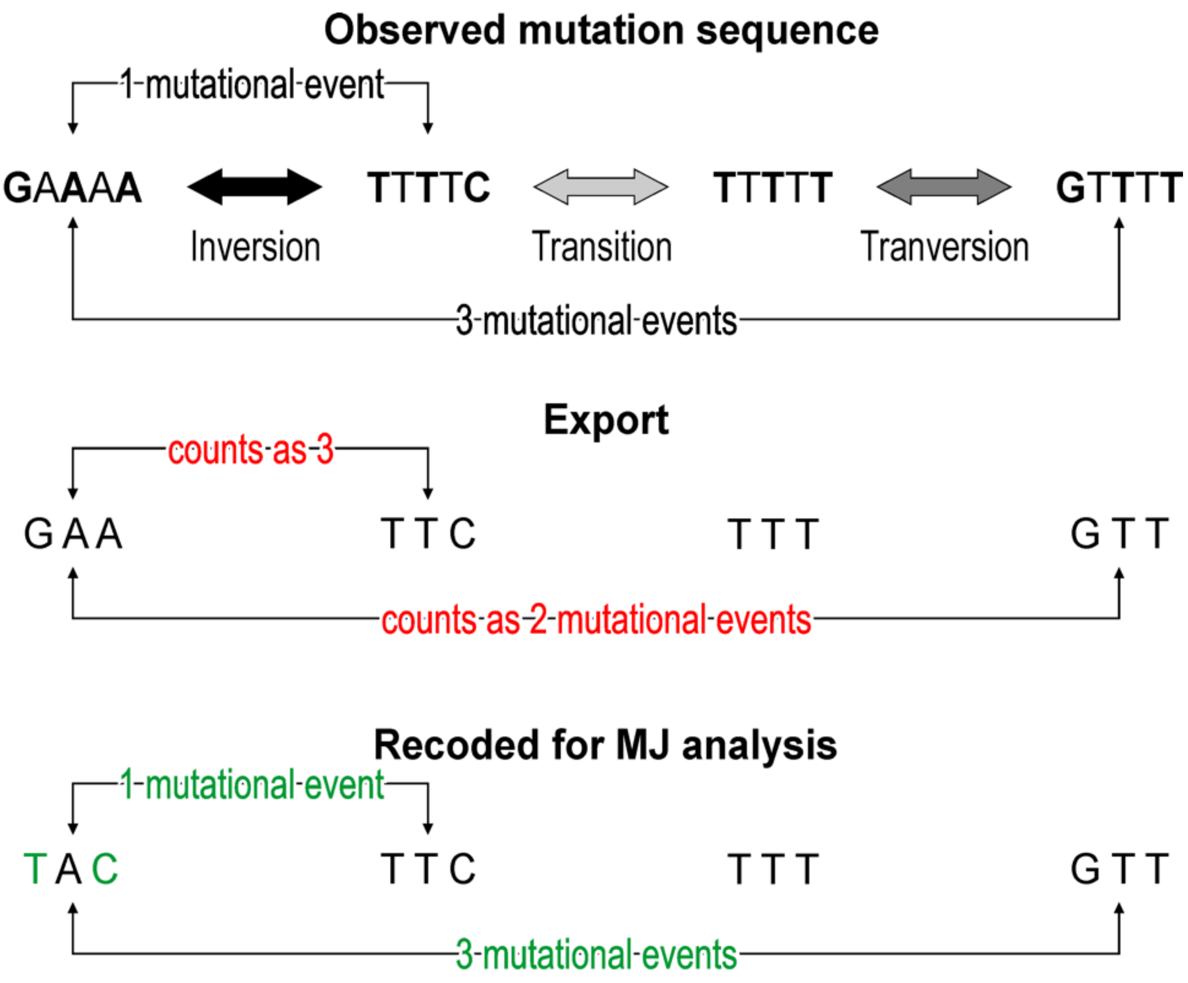

Figure S2 (A): **Examples of character re-coding**. Pseudo-loop motif (inversion with secondary point mutations), as found in the complementary ('pseudo-hairpin') region of the *trnK-rps16* in Clade III and IV (characters 7–9).

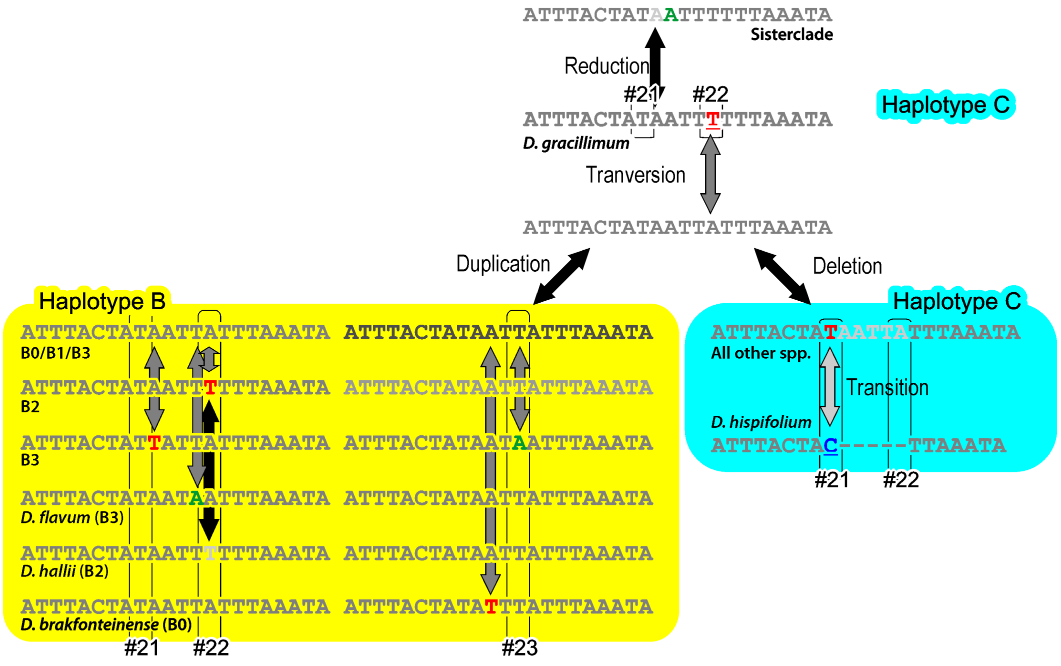

Oligonucleotide motif at 5' end of *trnS-trnG* in Clades 5 and 6 (chars # 21–23)

Figure S2 (B): **Examples of character re-coding**. Oligonucleotide motif at 5' end of *trnS-trnG* in Clades V and VI (characters 21–23).

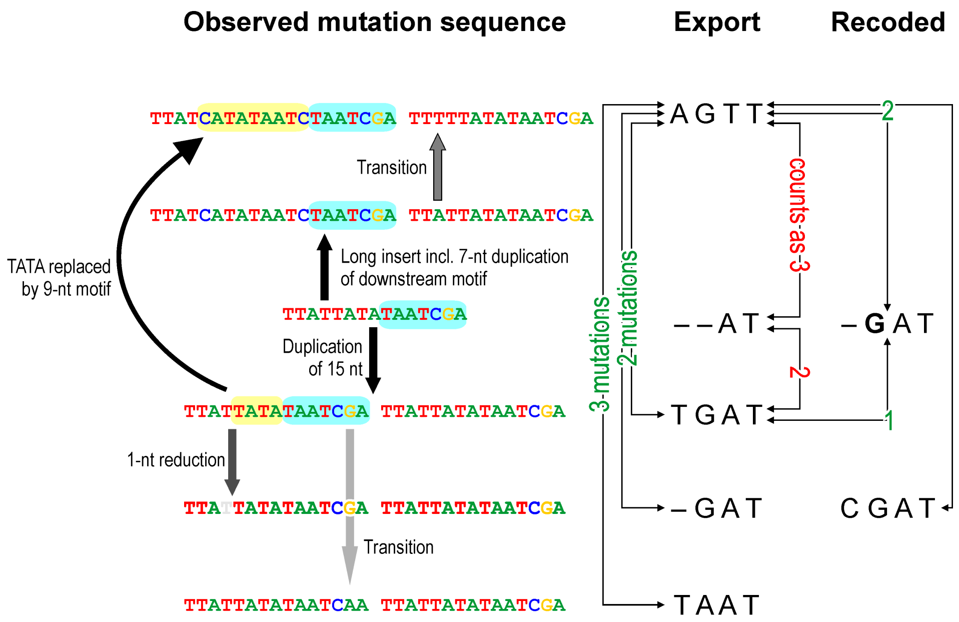

Indel pattern with secondary SNP in the centre of *trnS-trnG* in Clades 7 to 9 (chars # 13–16)

Figure S2 (C): **Examples of character re-coding**. Indel pattern with secondary SNP in the centre of *trnS-trnG* in Clades VII–IX (characters 13–16).
