# Supplementary material for: Phylogenetic relationships in the southern African genus Drosanthemum (Ruschioideae, Aizoaceae)
Source: PeerJ. 2020 May 8;8:e8999. doi: 10.7717/peerj.8999 (PMC7213013; doi:10.7717/peerj.8999)

Drosanthemum rDNA networks

Alastair Potts

16 Jul 2019

```
library(ape)
library(pegas)
library(dplyr)
library(ggplot2)
library(scales)
library(flextable)
```

SP network for ITS variants of (cp) clade I to IX

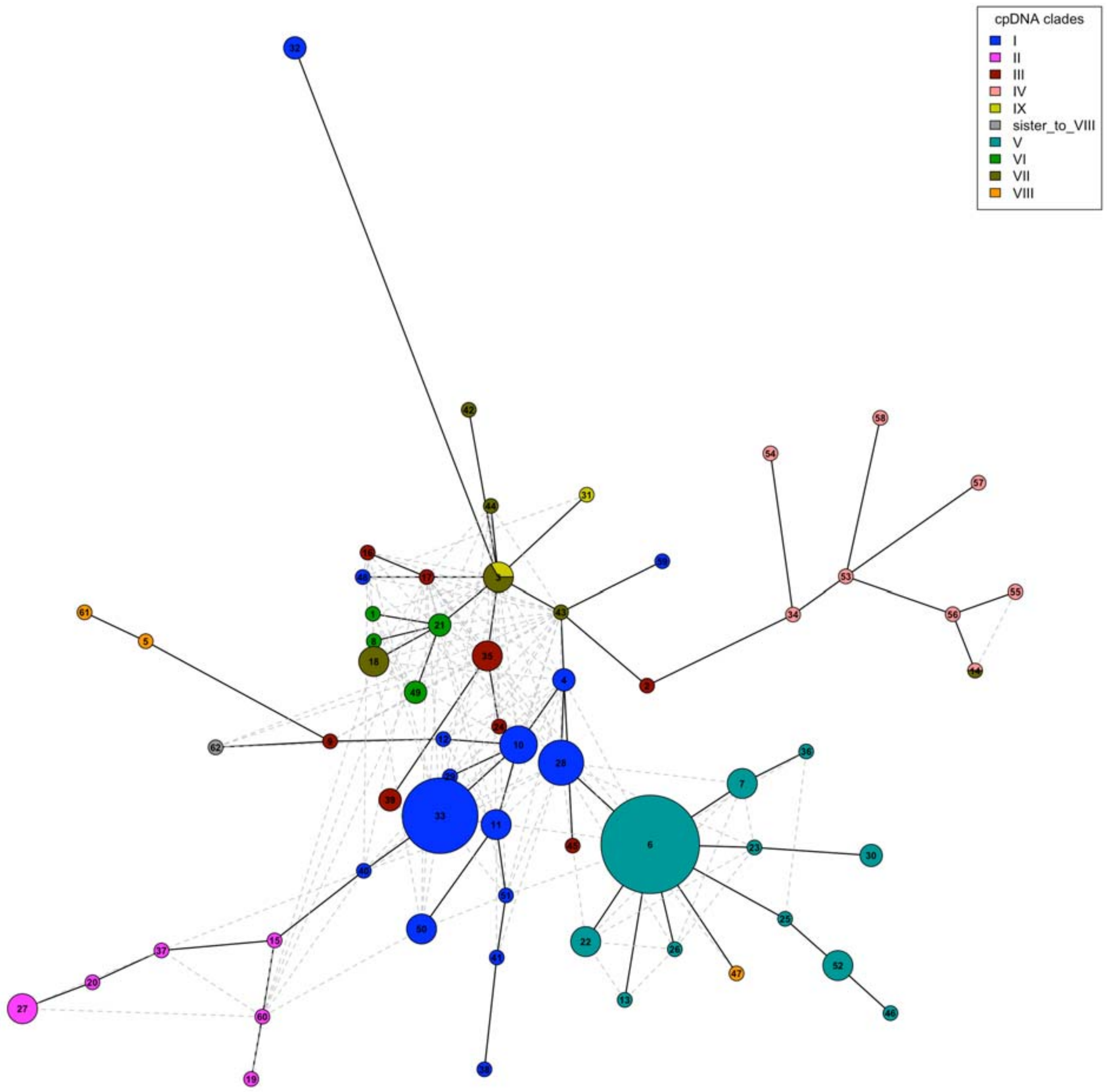

The star-shape is generally taken to be an indication of a bottleneck event with subsequent expansion (if we were looking at species phylogeography).

Given its central location, haplotype 3 might indicate the ancestral haplotype (rather than the common 6…)

ITS_hap	Accession
1	Drosanthemum_acuminatum_HH_34608
2	Drosanthemum_acutifolium_HH_32406
3	Drosanthemum_anemophilum_VanJaarsveld_sn
3	Drosanthemum_calycinum_HH_32211
3	Drosanthemum_papillatum_HH_34624
4	Drosanthemum_archeri_HH_31774
4	Drosanthemum_archeri_HH_31788
5	Drosanthemum_asperulum_HH_34502

ITS_hap	Accession
6	Drosanthemum_attenuatum_aff_HH_34650
6	Drosanthemum_attenuatum_HH_11826
6	Drosanthemum_austricola_HH_34695
6	Drosanthemum_boerhavii_HH_34697
6	Drosanthemum_cereale_HH_34491
6	Drosanthemum_chrysum_HH_34631
6	Drosanthemum_hallii_HH_34610
6	Drosanthemum_insolitum_HH_34596
6	Drosanthemum_pulchrum_HH_34712
6	Drosanthemum_stokoei_Bayer_7454
6	Drosanthemum_strictifolium_HH_34480
6	Drosanthemum_uniondalensis_HH_34813
7	Drosanthemum_aureopurpureum_HH_34478
7	Drosanthemum_brakfonteinii_HH_34688
7	Drosanthemum_micans_HH_34597
8	Drosanthemum_bicolor_HH_32459
9	Drosanthemum_brevifolium_HH_32242
10	Drosanthemum_candens_HH_25014
10	Drosanthemum_floribundum_HH_34403
10	Drosanthemum_hispidum_HH_33866
10	Drosanthemum_sp_Drosanthemum_HH_30568
11	Drosanthemum_candens_HH_30541
11	Drosanthemum_dejagerae_HH_31812
11	Drosanthemum_sp_Drosanthemum_HH_33182
12	Drosanthemum_candens_HH_31897
13	Drosanthemum_cereale_HH_34492
14	Drosanthemum_crassum_HH_25896
14	Drosanthemum_crassum_HH_25896
15	Drosanthemum_curtophyllum_HH_32673
16	Drosanthemum_cymiferum_HH_25811
17	Drosanthemum_cymiferum_HH_31686
18	Drosanthemum_cymiferum_HH_32250
18	Drosanthemum_expersum_HH_34590
18	Drosanthemum_sp_Quastea_HH_34598
19	Drosanthemum_delicatulum_HH_32462
20	Drosanthemum_dipageae_HH_34409
21	Drosanthemum_ecclesianum_HH_34814
21	Drosanthemum_ecclesianum_HH_34815
22	Drosanthemum_edwardsiae_HH_34648
22	Drosanthemum_lavisii_HH_34693
22	Drosanthemum_speciosum_HH_34619
23	Drosanthemum_edwardsiae_HH_34651
24	Drosanthemum_erigeriflorum_HH_26196
25	Drosanthemum_flammeum_HH_34460
26	Drosanthemum_flavum_HH_34706
27	Drosanthemum_fourcadei_HH_33900
27	Drosanthemum_nodosum_HH_34404
27	Drosanthemum_sp_Xamera_HH_34586
28	Drosanthemum_framesii_HH_32339

ITS_hap	Accession
28	Drosanthemum_glabrescens_HH_32256
28	Drosanthemum_marinum_HH_32205
28	Drosanthemum_prostratum_aff_HH_34316
28	Drosanthemum_prostratum_HH_34592
29	Drosanthemum_hispidum_HH_34262
30	Drosanthemum_hispifolium_HH_32206
30	Drosanthemum_hispifolium_HH_34587
31	Drosanthemum_inornatum_HH_32654
32	Drosanthemum_intermedium_HH_32200
32	Drosanthemum_intermedium_HH_32439
33	Drosanthemum_latipetalum_HH_31568
33	Drosanthemum_latipetalum_HH_31605
33	Drosanthemum_latipetalum_HH_32217
33	Drosanthemum_muirii_HH_30321
33	Drosanthemum_nordenstamii_HH_31533
33	Drosanthemum_oculatum_HH_31710
33	Drosanthemum_schoenlandianum_HH_25751
33	Drosanthemum_sp_Drosanthemum_HH_32218
33	Drosanthemum_subplanum_HH_32259
34	Drosanthemum_lique_HH_34447
35	Drosanthemum_luederitzii_HH_25972
35	Drosanthemum_luederitzii_HH_26095
35	Drosanthemum_obibense_HH_25979
36	Drosanthemum_micans_LeRoux_83_2
37	Drosanthemum_montaguense_HH_31824
38	Drosanthemum_muiri_HH_32209
39	Drosanthemum_nollothense_HH_31547
39	Drosanthemum_nollothense_HH_31569
40	Drosanthemum_oculatum_HH_31680
41	Drosanthemum_opacum_HH_32212
42	Drosanthemum_papillatum_HH_34456
43	Drosanthemum_papillatum_HH_34607
44	Drosanthemum_papillatum_HH_34614
45	Drosanthemum_parvifolium_HH_30399
46	Drosanthemum_pustulatum_HH_34698
47	Drosanthemum_quadratum_HH_34503
48	Drosanthemum_schoenlandianum_Bruyns_7172
49	Drosanthemum_semiglobosum_HH_34593
49	Drosanthemum_thudichumii_HH_34714
50	Drosanthemum_sp_Drosanthemum_HH_25365
50	Drosanthemum_sp_Drosanthemum_HH_32392neu
50	Drosanthemum_sp_Drosanthemum_HH_34496
51	Drosanthemum_sp_Drosanthemum_HH_34472
52	Drosanthemum_sp_Ossicula_HH_34699
52	Drosanthemum_striatum_HH_29013
52	Drosanthemum_striatum_HH_34720
53	Drosanthemum_sp_Vespertina_HH_26170a
54	Drosanthemum_sp_Vespertina_HH_31059
55	Drosanthemum_sp_Vespertina_HH_32378

ITS_hap	Accession
56	Drosanthemum_sp_Vespertina_HH_32404
57	Drosanthemum_sp_Vespertina_HH_33490
58	Drosanthemum_sp_Vespertina_HH_34804
59	Drosanthemum_sp_Vespertina_Mucina_161005_14
60	Drosanthemum_subclausum_HH_25737
61	Drosanthemum_tetramerum_HH_34488
62	Drosanthemum_zygophylloides_Mucina_130216_4

Mismatch distribution

The histogram shows the observed distribution (frequencies) of pairwise distances from the set of ITS sequences. The lines show an empirical density estimate (in blue) and the expected distribution under stable population (Rogers and Harpending 1992).

```
MMD(as.DNAbin(nex))
```

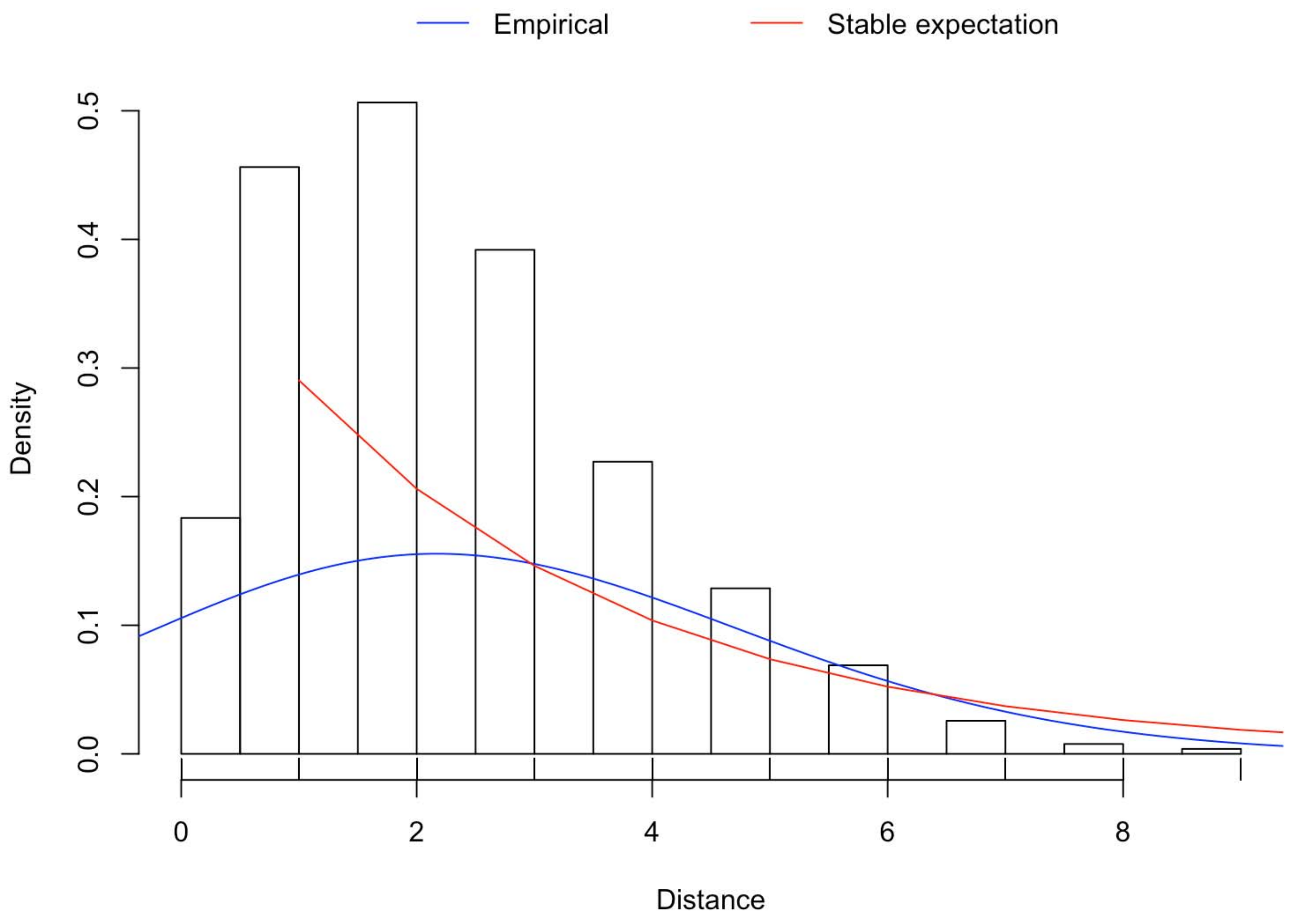

The mismatch distribution indicates an expanding “population”, in this case, probably radiations/emeregence of new species.

Subclade networks

SP network for ITS variants of clade I to IV, VIII, and IX

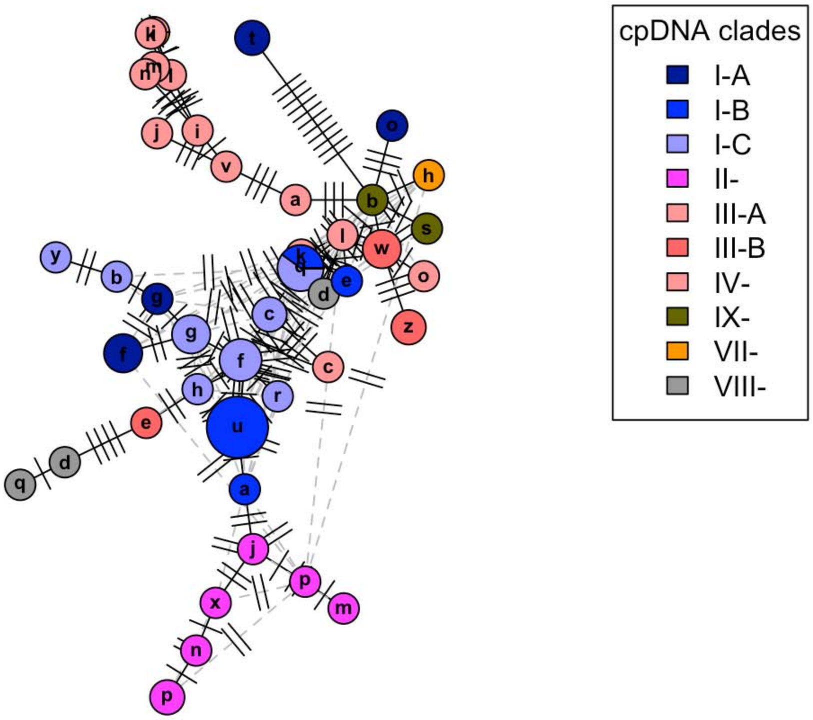

SP network for ITS variants of clade I to IV

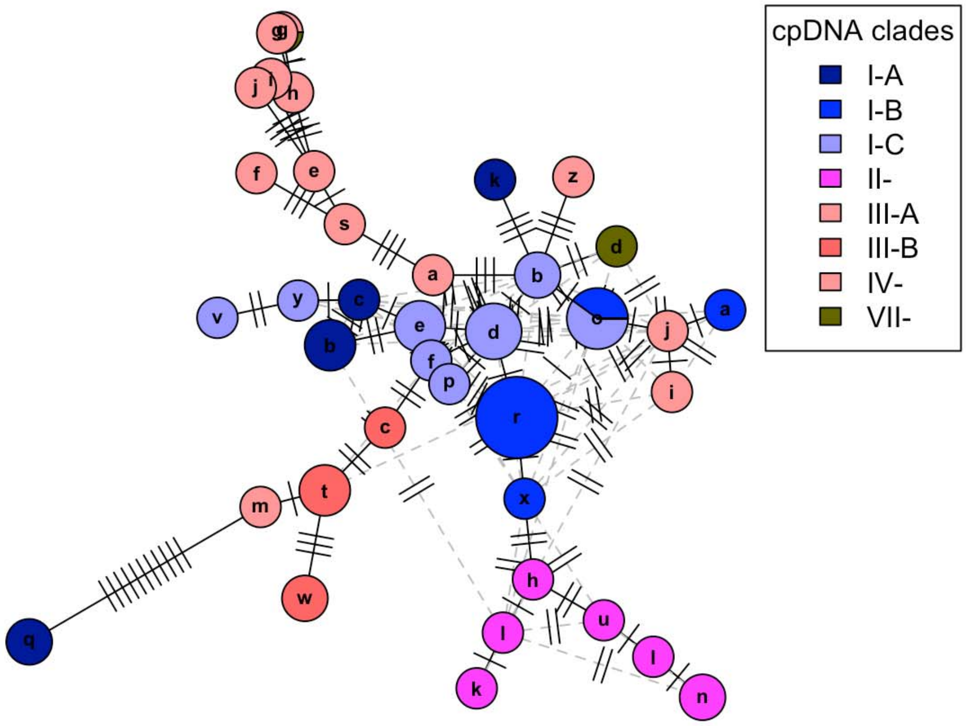

SP network for ITS variants of clade I and II

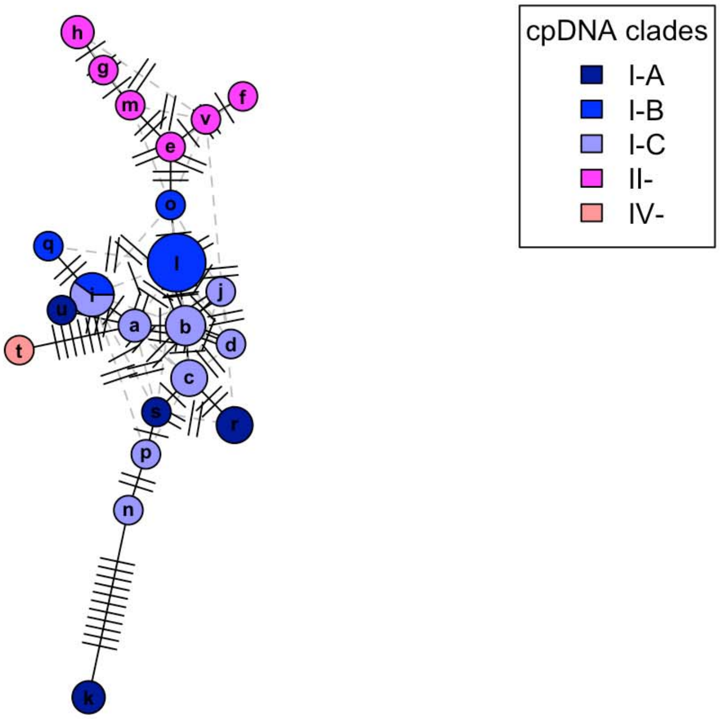

SP network for ITS variants of clade III and IV

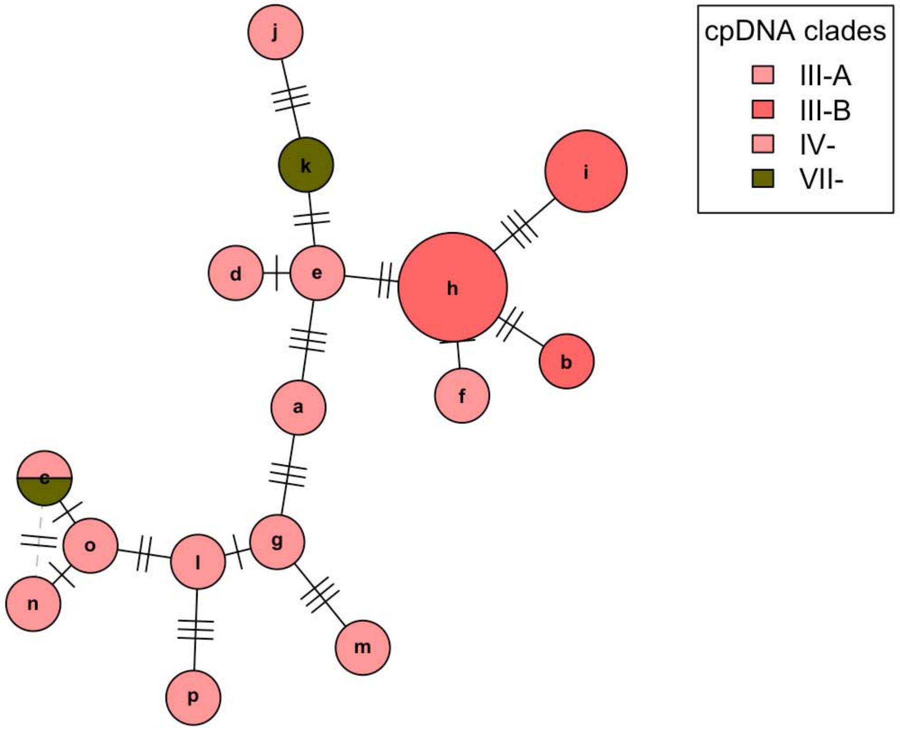

SP network for ITS variants of clade V to VII

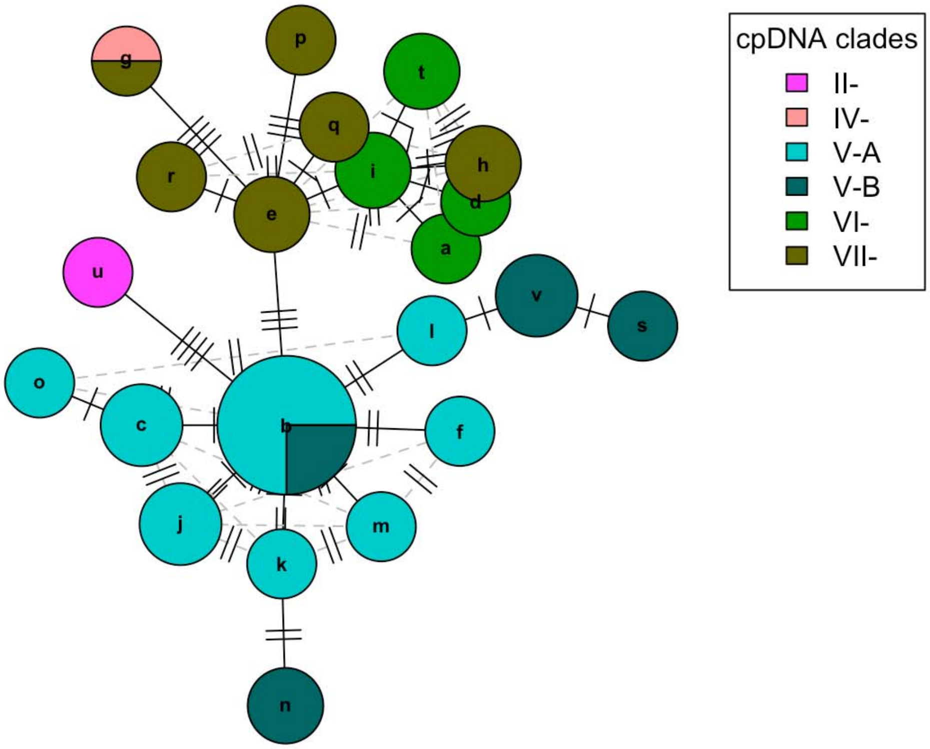

SP network for ITS variants of clade VIII, sister to VIII, and IX

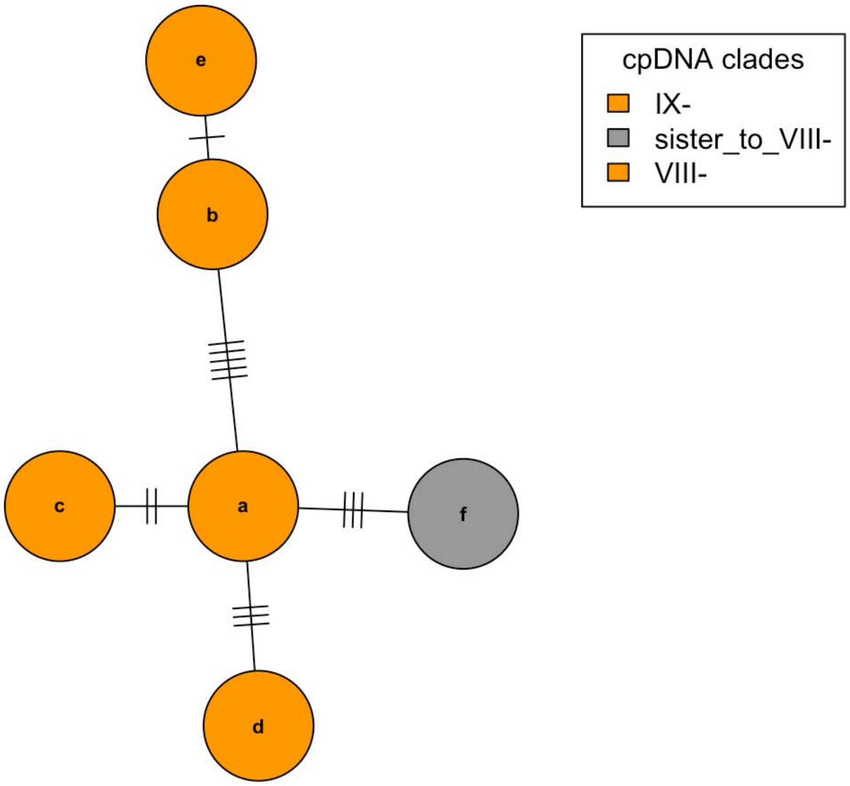

Supplement: Supplemental Information 3 [file peerj-08-8999-s003.pdf]
