# Supplementary material for: Phylogenetic relationships in the southern African genus Drosanthemum (Ruschioideae, Aizoaceae)
Source: PeerJ. 2020 May 8;8:e8999. doi: 10.7717/peerj.8999 (PMC7213013; doi:10.7717/peerj.8999)
Supplement: Supplemental Information 4 — Results of outgroup-EPA detailing the five most probable root positions in Drosanthemum (Figs. S4.2–6), and the probability of all query placements (Table S4). Also including the result of the phylogenetic ML analysis of the “Ruschioideae” cpDNA data (Fig. S4.1) [file peerj-08-8999-s004.pdf]

Supplementary information S4

to: Phylogenetic relationships in the southern African genus *Drosanthemum* (Ruschioideae, Aizoaceae)

by Liede-Schumann, Grimm, Nürk, Potts, Meve & Hartmann

Contains:

1) Results of maximum likelihood analysis of the partitioned "Ruschioideae" cpDNA dataset (Fig. S4.1).
2) Results of outgroup-EPA analyses (Berger, Krompass & Stamatakis 2011) detailing the five potential root positions in *Drosanthemum* with queried Ruschieae outgroup taxa supporting a respective scenario (Figs. S4.2–6). Likelihood weight ratios for the queried outgroup taxa and probability estimates (Table S4). Raw data, code and full analysis output for all partitioning schemes and marker combinations are available at Dryad, Liede-Schumann et al. 2019.

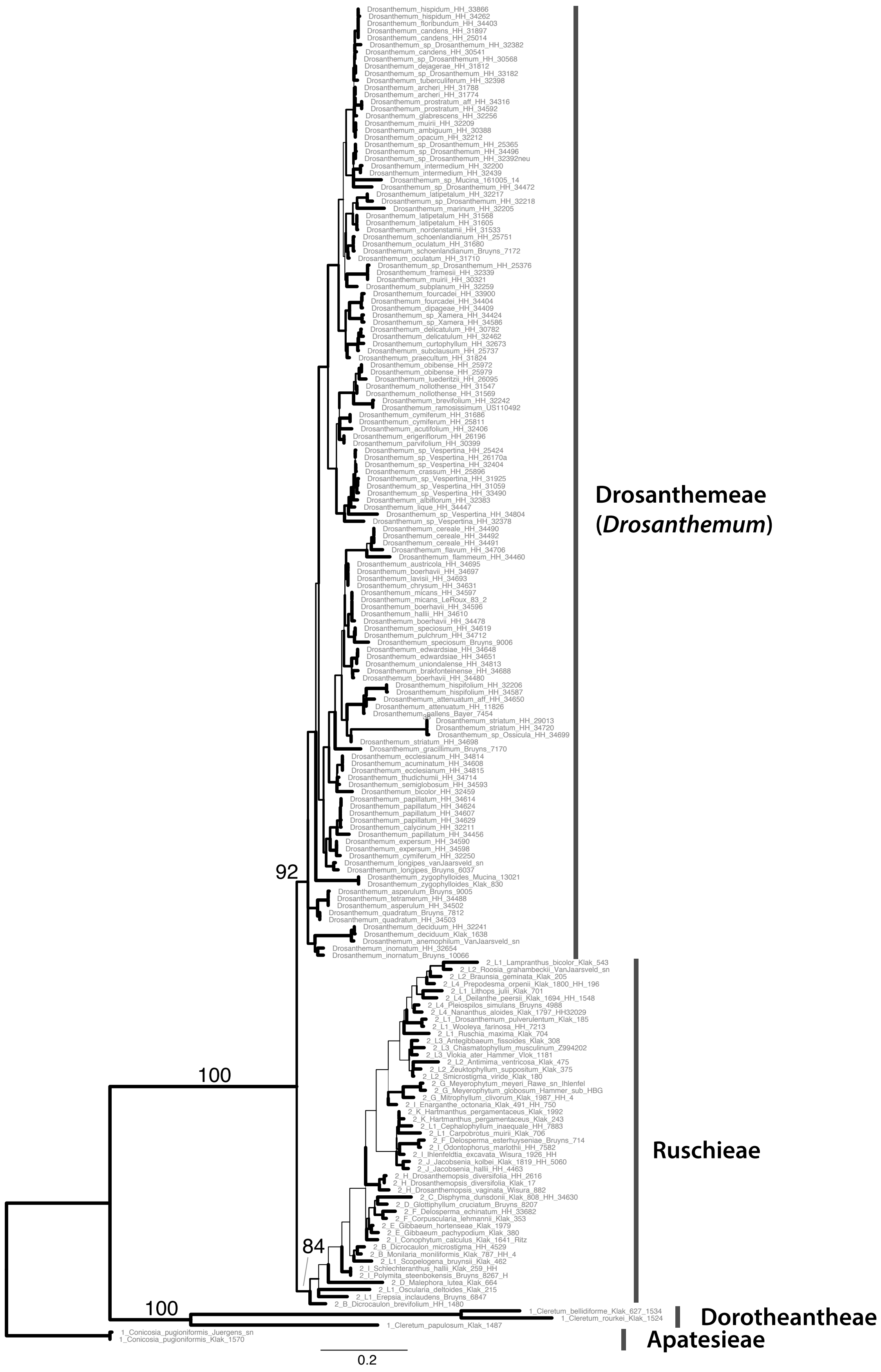

Figure S4.1: **Phylogeny Ruschioideae.** ML tree inferred by partitioned analysis of the cpDNA sequence data for Ruschioideae. The tribes of Ruschioideae are annotated.

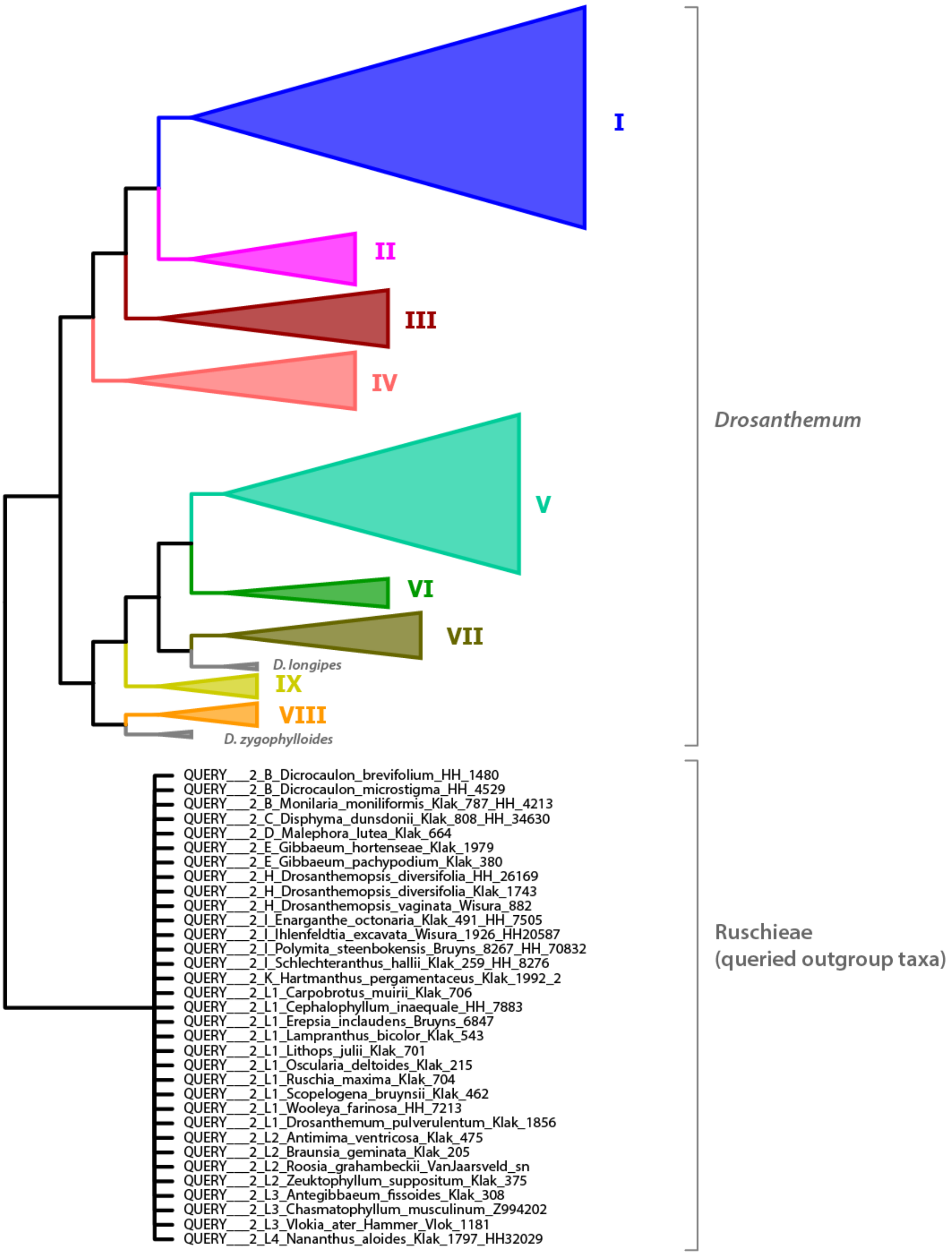

Figure S4.2: ***Drosanthemum* root position scenario 1**. Mean probability estimate 0.260.

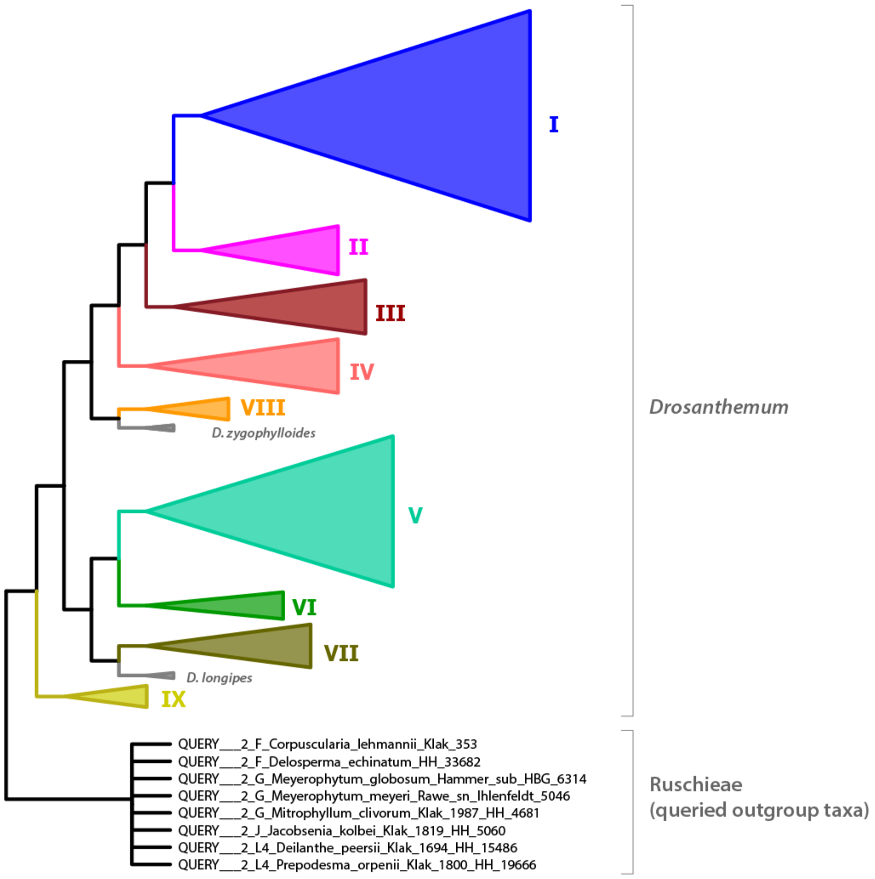

Figure S4.3: ***Drosanthemum* root position scenario 2**. Mean probability estimate 0.232.

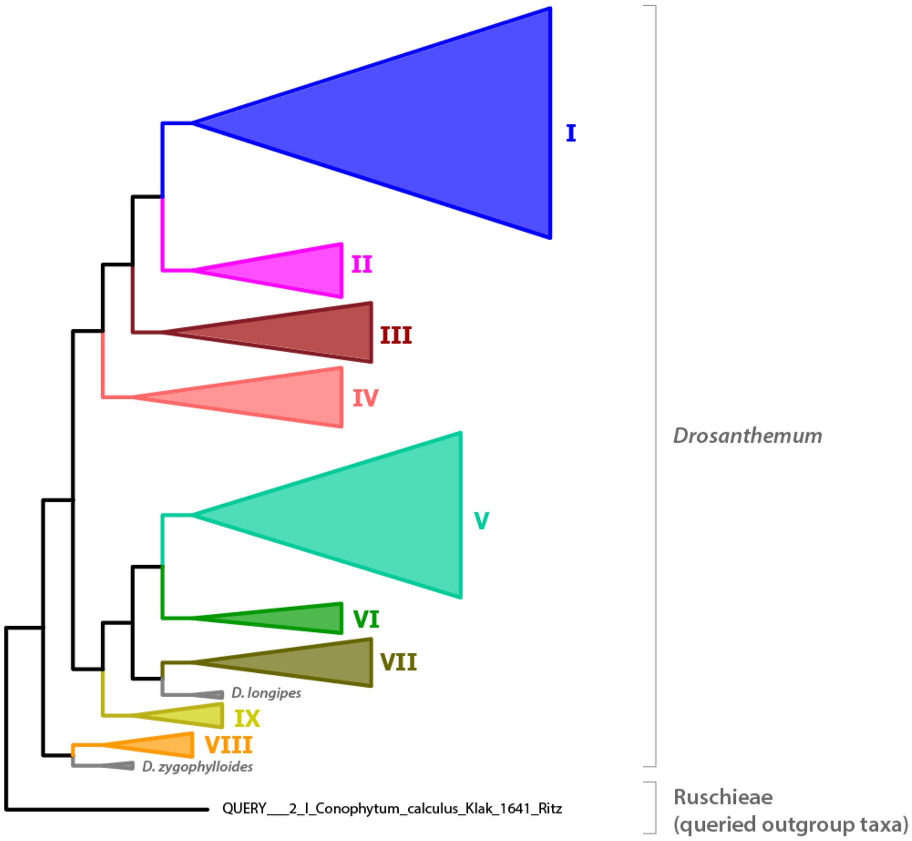

Figure S4.4: ***Drosanthemum* root position scenario 3**. Mean probability estimate 0.143.

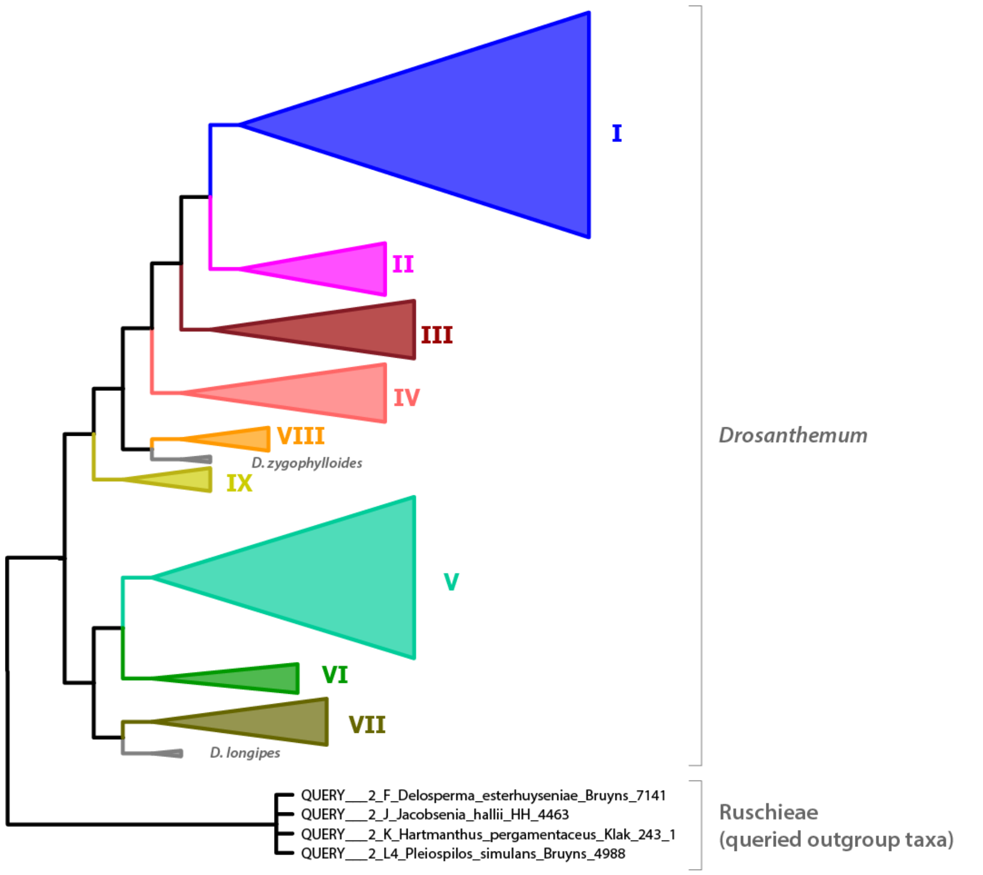

Figure S4.5: ***Drosanthemum* root position scenario 4**. Mean probability estimate 0.138.

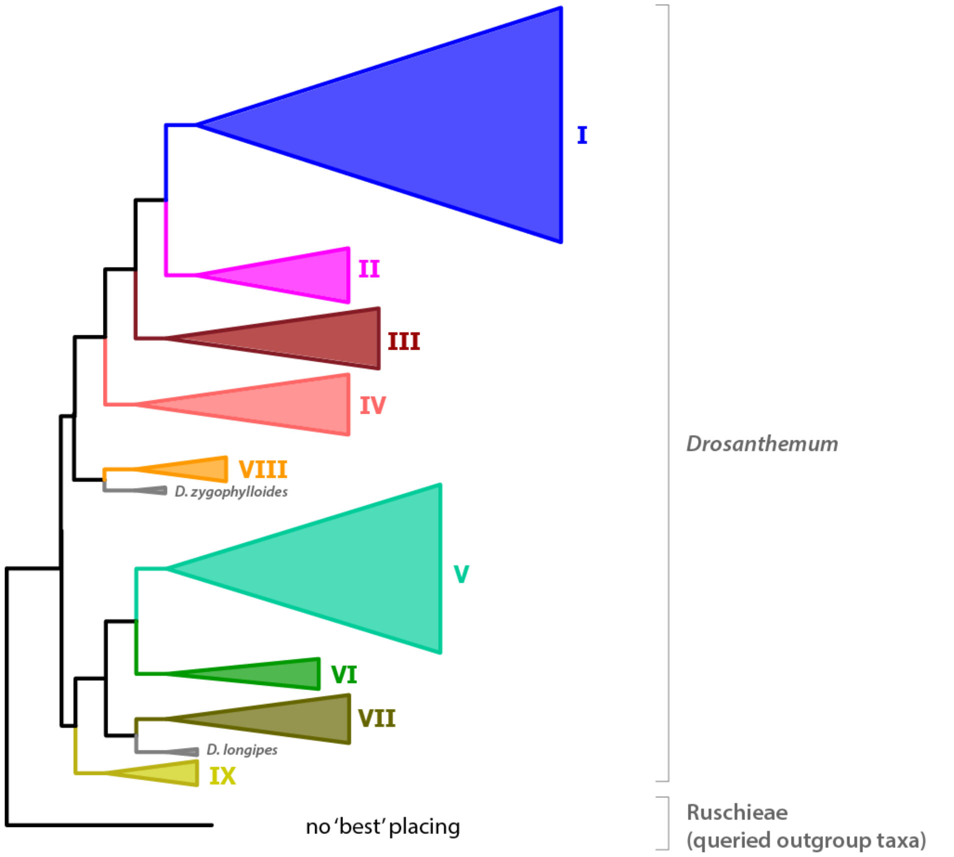

Figure S4.6: ***Drosanthemum* root position scenario 5**. Mean probability estimate 0.137.

Table S4. Results of outgroup EPA. *Drosanthemum* rooting scenarios S1–S11 detailing inferred root position by node numbers, number of queried outgroup taxa supporting a scenario, the mean probability estimates and the queried outgroup taxa by their likelihood weight ratio per scenario. Numbers in bold fond indicate the supporting taxa per scenario (i.e. highest likelihood weight ratio).

**Query results**	**S 1**	**S 2**	**S 3**	**S 4**	**S 5**	**S 6**	**S 7**	**S 8**	**S 9**	**S 10**	**S 11**
Node № [a]	180	182	232	186	181	233	234	223	222	187	224
Internal node № [b]	I94	I96	I105	I198	I95	I199	I202	I178	I177	I106	I181
# placings (supporting taxa)	33	8	1	4	0	2	0	0	1	0	0
Mean probability estimate	0.2596	0.2321	0.1430	0.1377	0.1372	0.0297	0.0192	0.0165	0.0102	0.0016	0.0010
Query taxa											
2_B_Dicrocaulon_brevifolium_HH_1480	**0.1789**	0.1777	0.1775	0.1781	0.1777	0.0162				0.0599	
2_B_Dicrocaulon_microstigma_HH_4529	**0.1932**	0.1915	0.1915	0.1917	0.1916	0.0175	0.0158				
2_B_Monilaria_moniliformis_Klak_787_HH_4213	**0.1910**	0.1895	0.1894	0.1898	0.1896	0.0175	0.0160				
2_C_Disphyma_dunsdonii_Klak_808_HH_34630	**0.7077**	0.0655	0.0655	0.0655	0.0655	0.0190					
2_D_Glottiphyllum_cruciatum_Bruyns_8207_HH_8809	0.0167			0.0170	0.0164			0.3408	**0.5002**	0.0182	0.0471
2_D_Malephora_lutea_Klak_664	**0.1831**	0.1816	0.1816	0.1817	0.1817	0.0195		0.0416			
2_E_Gibbaeum_hortenseae_Klak_1979	**0.1927**	0.1907	0.1908	0.1909	0.1908	0.0197	0.0157				
2_E_Gibbaeum_pachypodium_Klak_380	**0.1923**	0.1899	0.1899	0.1904	0.1899	0.0174	0.0157				
2_F_Corpuscularia_lehmannii_Klak_353	0.0751	**0.6587**	0.0746	0.0747	0.0746	0.0224	0.0083				
2_F_Delosperma_echinatum_HH_33682	0.0627	**0.7306**	0.0622	0.0624	0.0623						
2_F_Delosperma_esterhuyseniae_Bruyns_7141	0.1900	0.1898	0.1899	**0.1900**	0.1899	0.0211	0.0175				
2_G_Meyerophytum_globosum_Hammer_sub_HBG_6314	0.1046	**0.5569**	0.1041	0.1042	0.1042	0.0100	0.0093				
2_G_Meyerophytum_meyeri_Rawe_sn_Ihlenfeldt_5046	0.0951	**0.5368**	0.0942	0.0943	0.0943	0.0720	0.0083				
2_G_Mitrophyllum_clivorum_Klak_1987_HH_4681	0.0949	**0.5406**	0.0952	0.0945	0.0944	0.0654	0.0089				
2_H_Drosanthemopsis_diversifolia_HH_26169	**0.1811**	0.1794	0.1794	0.1795	0.1794	0.0731	0.0169				
2_H_Drosanthemopsis_diversifolia_Klak_1743	**0.1913**	0.1894	0.1894	0.1896	0.1895	0.0190	0.0180				
2_H_Drosanthemopsis_vaginata_Wisura_882	**0.1911**	0.1893	0.1893	0.1894	0.1893	0.0188	0.0182				
2_I_Conophytum_calculus_Klak_1641_Ritz	0.1902	0.1898	**0.1906**	0.1899	0.1898	0.0208	0.0167				
2_I_Enarganthe_octonaria_Klak_491_HH_7505	**0.8948**	0.0250	0.0250	0.0250	0.0250						
2_I_Ihlenfeldtia_excavata_Wisura_1926_HH20587	**0.8523**	0.0349	0.0349	0.0349	0.0349						
2_I_Odontophorus_marlothii_HH_7582	0.0464		0.2988			**0.3494**	0.2969				
2_I_Polymita_steenbokensis_Bruyns_8267_HH_70832	**0.8817**	0.0278	0.0278	0.0279	0.0279						

**Query results**	**S 1**	**S 2**	**S 3**	**S 4**	**S 5**	**S 6**	**S 7**	**S 8**	**S 9**	**S 10**	**S 11**
2_I_Schlechteranthus_hallii_Klak_259_HH_8276	**0.1964**	0.1857	0.1857	0.1859	0.1858	0.0284	0.0184				
2_J_Jacobsenia_hallii_HH_4463	0.1894	0.1878	0.1879	**0.2011**	0.1879	0.0211	0.0192				
2_J_Jacobsenia_kolbei_Klak_1819_HH_5060	0.0475	**0.7941**	0.0470	0.0471	0.0471	0.0100					
2_K_Hartmanthus_pergamentaceus_Klak_1992_2	**0.1925**	0.1908	0.1908	0.1909	0.1909	0.0206	0.0155				
2_K_Hartmanthus_pergamentaceus_Klak_243_1	0.1876	0.1871	0.1871	**0.1919**	0.1871	0.0236	0.0210				
2_L1_Carpobrotus_muirii_Klak_706	**0.1922**	0.1905	0.1905	0.1907	0.1906	0.0212	0.0158				
2_L1_Cephalophyllum_inaequale_HH_7883	**0.1799**	0.1785	0.1785	0.1787	0.1786	0.0796	0.0162				
2_L1_Drosanthemum_pulverulentum_Klak_1856	**0.1903**	0.1899	0.1899	0.1900	0.1900	0.0198	0.0175				
2_L1_Erepsia_inclaudens_Bruyns_6847	**0.1902**	0.1886	0.1886	0.1888	0.1887	0.0201	0.0189				
2_L1_Lampranthus_bicolor_Klak_543	**0.4983**	0.0251	0.0251	0.0251	0.0251			0.3913			
2_L1_Lithops_julii_Klak_701	**0.1922**	0.1904	0.1904	0.1906	0.1905	0.0196	0.0163				
2_L1_Oscularia_deltoides_Klak_215	**0.1916**	0.1896	0.1896	0.1897	0.1897	0.0186	0.0178				
2_L1_Ruschia_maxima_Klak_704	**0.1842**	0.1826	0.1826	0.1828	0.1827	0.0175		0.0371			
2_L1_Scopelogena_bruynsii_Klak_462	**0.1907**	0.1899	0.1899	0.1901	0.1900	0.0207	0.0170				
2_L1_Wooleya_farinosa_HH_7213	**0.1931**	0.1916	0.1916	0.1917	0.1916	0.0174	0.0163				
2_L2_Antimima_ventricosa_Klak_475	**0.1922**	0.1903	0.1903	0.1905	0.1904	0.0193	0.0165				
2_L2_Braunsia_geminata_Klak_205	**0.8531**	0.0344	0.0344	0.0345	0.0345						
2_L2_Roosia_grahambeckii_VanJaarsveld_sn	**0.7860**	0.0506	0.0507	0.0507	0.0507						
2_L2_Smicrostigma_viride_Klak_180	0.1356	0.1344	0.1346	0.1346	0.1345	**0.1882**	0.1318				
2_L2_Zeuktophyllum_suppositum_Klak_375	**0.7438**	0.0597	0.0597	0.0598	0.0598						
2_L3_Antegibbaeum_fissoides_Klak_308	**0.1921**	0.1904	0.1904	0.1905	0.1905	0.0188	0.0167				
2_L3_Chasmatophyllum_musculinum_Z994202	**0.1911**	0.1907	0.1907	0.1909	0.1908	0.0181	0.0182				
2_L3_Vlokia_ater_Hammer_Vlok_1181	**0.1919**	0.1900	0.1900	0.1902	0.1901	0.0214	0.0168				
2_L4_Deilanthe_peersii_Klak_1694_HH_15486	0.0108	**0.9546**	0.0107	0.0107	0.0107						
2_L4_Nananthus_aloides_Klak_1797_HH32029	**0.1921**	0.1903	0.1903	0.1904	0.1903	0.0180	0.0184				
2_L4_Pleiospilos_simulans_Bruyns_4988	0.1901	0.1900	0.1900	**0.1908**	0.1900	0.0211	0.0175				
2_L4_Prepodesma_orpenii_Klak_1800_HH_19666	0.1188	**0.5005**	0.1177	0.1178	0.1177	0.0115	0.0107				

a, Node No refers to the ML cpDNA tree (Fig 3), see Dryad Liede-Schumann et al. 2019, file d_RAxML_bestTree.6_cp.q.tre (can be viewed in e.g. R using ape::nodelabels).
b, Internal node No refers to the RAxML EPA output file detailing position of queried outgroup taxa, see Dryad Liede-Schumann et al. 2019, file RAxML_labelledTree.OGPA.q (can be viewed in e.g. Dendroscope).
